# Machine Learning-Driven Design of Fluorescent Materials: Principles, Methodologies, and Future Directions

**DOI:** 10.3390/nano15191495

**Published:** 2025-09-30

**Authors:** Qihang Bian, Xiangfu Wang

**Affiliations:** 1 College of Electronic and Optical Engineering & College of Flexible Electronics (Future Technology), Nanjing University of Posts and Telecommunications, Nanjing 210023, China; b22020506@njupt.edu.cn; 2 Key Laboratory of Radio Frequency and Micro-Nano Electronics of Jiangsu Province, Nanjing 210023, China

**Keywords:** machine learning, fluorescent materials, physics-informed learning, inverse design

## Abstract

Dual-mode fluorescent materials are vital in bioimaging, sensing, displays, and lighting, owing to their efficient emission of visible or near-infrared light. Traditional optimization methods, including empirical experiments and quantum chemical computations, suffer from high costs, high labor intensities, and difficulties capturing complex relationships among molecular structures, synthesis parameters, and key photophysical properties. In this review, fundamental principles, key methodologies, and representative applications of machine learning (ML) in predicting fluorescent material performance are systematically summarized. The core ML techniques covered include supervised regression, neural networks, and physics-informed hybrid frameworks. The representative fluorescent materials analyzed encompass aggregation-induced emission (AIE) luminogens, thermally activated delayed fluorescence (TADF) emitters, quantum dots, carbon dots, perovskites, and inorganic phosphors. This review details the modeling approaches and typical workflows—such as data preprocessing, descriptor selection, and model validation—and highlights algorithmic optimization strategies such as data augmentation, physical constraints embedding, and transfer learning. Finally, prevailing challenges, including limited high-quality data availability, weak model interpretability, and insufficient model transferability, are discussed.

## 1. Introduction

Fluorescent materials have become foundational elements across numerous advanced technological domains, significantly influencing biomedical imaging, optical sensing, display technologies, and solid-state lighting. For instance, fluorescent probes have profoundly transformed biomedical imaging by enabling high-resolution, real-time visualization of cellular and molecular processes [1,2,3]. Likewise, quantum dot-based displays and organic light-emitting diodes (OLEDs) represent revolutionary advances in consumer electronics, greatly improving color purity, display efficiency, and energy consumption [4,5]. Additionally, fluorescent-based sensors exhibit superior sensitivity and selectivity in environmental monitoring, providing robust analytical solutions for detecting contaminants and pollutants [6,7,8,9]. However, despite substantial progress, the ongoing demand for novel fluorescent materials featuring enhanced emission efficiency, spectral tunability, and stability under diverse operating conditions continues to propel intensive research and innovation at the intersection of materials science, photonics, and biotechnology [10,11,12]. The significant economic impact of these applications is underscored by substantial market valuations and robust growth projections. According to data from QYResearch in 2025, the global market for OLED blue fluorescent materials, crucial for displays, was valued at USD 404 million in 2023 and is expected to rise to USD 851 million by 2030, exhibiting a compound annual growth rate of 10.8%. China National Chemical Information Center believes that, by 2025, the dominant force in China’s fluorescent material supply chain will have an overall market size of CNY 35 billion (approximately USD 4.8 billion), driven by LED lighting sectors (58%), and is expected to expand to CNY 65 billion (approximately USD 9 billion) by 2030. In addition, according to MarketsandMarkets’ data in 2024, the global biomarker market with fluorescent materials accounting for over 30% is expected to reach USD 7.8 billion by 2025, driven by advances in medical diagnosis and imaging. This substantial and growing economic potential further incentivizes the development of high-performance fluorescent materials.

Traditional approaches for discovering and optimizing fluorescent materials primarily rely on empirical trial-and-error experiments and first-principles computational simulations, particularly density functional theory (DFT) [13,14,15]. Experimental techniques, although valuable, often face substantial constraints due to high costs, labor-intensive processes, and lengthy time requirements, limiting their scalability, efficiency, and reproducibility [16,17]. Similarly, theoretical approaches, including DFT and time-dependent DFT (TD-DFT), while adept at elucidating electronic and excited-state properties, demand considerable computational resources, making exhaustive exploration of extensive molecular design spaces impractical [13,15,18]. Moreover, these conventional methods struggle to effectively capture the inherently complex and nonlinear relationships among molecular structures, synthesis conditions, and fluorescence properties, thereby significantly impeding the systematic and rapid identification of high-performance materials [13,16]. Thus, there exists an imperative need for alternative methodologies capable of efficiently and systematically exploring vast chemical spaces.

Recently, machine learning (ML) has emerged as a powerful alternative, effectively addressing the intrinsic limitations of conventional approaches by utilizing large datasets to rapidly and accurately predict fluorescent properties [19,20,21]. ML methods, such as neural networks, support vector machines, and ensemble learning algorithms, directly extract intricate, nonlinear relationships between structural descriptors and fluorescence performance, circumventing explicit quantum mechanical modeling and significantly reducing computational resources and time [20,21,22]. Recent applications have demonstrated ML’s robust capability to predict essential photophysical properties—including emission wavelength, quantum yield, and operational stability—in diverse fluorescent systems such as aggregation-induced emission (AIE) luminogens, thermally activated delayed fluorescence (TADF) emitters, quantum dots, and perovskite-based materials [23,24,25,26]. Nonetheless, a systematic and comprehensive integration of ML methodologies, comparative analyses, and their application across different fluorescent material categories remains sparse.

**Figure 1:**
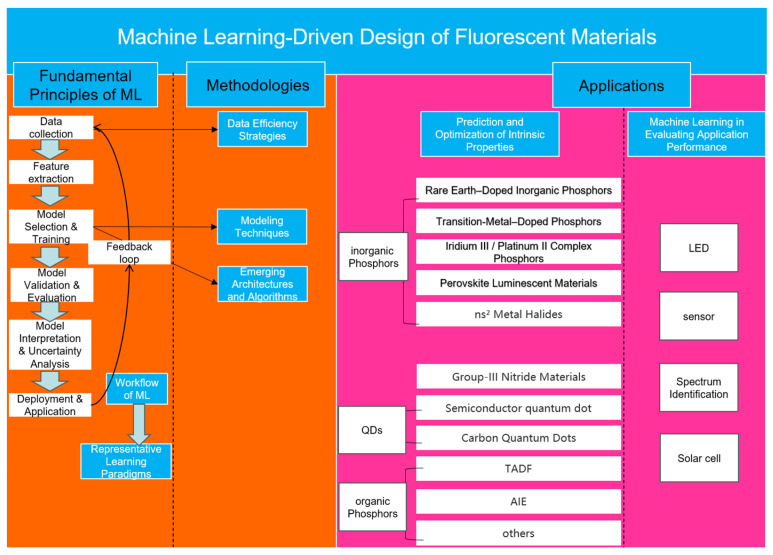
The framework structure and relationships between the different sections of this overview.

Addressing this critical gap, the present review systematically summarizes the foundational methods, fundamental principles, and advanced applications of ML in fluorescent material research, as shown in Figure 1. Initially, we detail the core ML-driven property prediction workflow, including data acquisition, feature engineering, model development, and validation. Subsequently, the review analyzes essential methodological frameworks, differentiating purely data-driven strategies from physics-informed approaches, while also emphasizing advanced ML techniques such as active learning and transfer learning. Following this, we critically survey representative ML applications across multiple classes of fluorescent materials—including AIE compounds, TADF emitters, quantum dots, carbon dots, metal-halide perovskites, and rare-earth-doped phosphors—highlighting unique challenges and solutions pertinent to each category. Finally, we synthesize comparative insights across material categories, identify prevailing research limitations such as data scarcity, interpretability challenges, and limited generalizability [20,27], and propose strategic directions for future research. These include integrating domain-specific physical knowledge and developing robust, multimodal ML frameworks to substantially advance fluorescent material design and discovery [19,28].

This review differs from previous studies in three key aspects. First, it provides a comprehensive overview covering a wide range of fluorescent materials, spanning from organic to inorganic materials. Second, it specifically introduces state-of-the-art research methodologies in cutting-edge fields, such as data mining using large language models and physics-informed neural networks. Finally, it offers a unique interdisciplinary and systematic comparative perspective, linking fundamental ML principles with specific applied case studies. This research approach provides timely and comprehensive guidance of this field, facilitating researchers in the discipline to quickly grasp the current state of cutting-edge developments.

## 2. Fundamental Principles of Machine Learning in Fluorescent Materials

### 2.1. Workflow of ML for Fluorescent Property Prediction

A typical machine learning (ML) workflow for predicting fluorescent material properties involves several interrelated stages that each influence the accuracy and robustness of the final model. These stages generally include data collection, feature extraction, model development, model validation, and final deployment for property prediction. In the data collection phase, researchers assemble datasets of fluorescence properties (e.g., emission wavelengths, photoluminescence quantum yields, lifetimes, stability metrics) from experiments, simulations, or databases. Ensuring that this data is high-quality and representative is critical, because errors or biases introduced at this stage can mislead the ML algorithms. It is often necessary to preprocess raw data (for example, handling missing or inconsistent entries) to improve reliability. Given the growing availability of public fluorescent materials datasets, careful curation and error-checking are essential to avoid propagating experimental noise into model training.

After gathering data, the next step is feature extraction (or featurization), where raw inputs are translated into structured descriptors capturing the underlying chemistry or physics of fluorescence. Depending on the problem, features may include physicochemical properties, structural fingerprints (such as molecular graphs or fragment-based descriptors), or even spectral characteristics. For example, predicting emission wavelength might involve features related to molecular conjugation length or electronic transition energies, whereas quantum yield prediction might use descriptors reflecting molecular rigidity or excited-state dynamics. The choice of representation strongly affects model performance—appropriate features allow for the algorithm to more easily discover meaningful patterns. Domain knowledge can guide feature design (e.g., using known photophysical parameters), although modern deep learning models can also learn abstract representations directly from data, bypassing manual feature engineering.

The growing importance of data-driven research has spurred the development of several publicly available databases and resources. These repositories are invaluable for benchmarking ML models, pre-training, and discovering new fluorescent materials. Table 1 summarizes a selection of key resources relevant to the community.

In the model development stage, an ML algorithm (or a combination of algorithms) is selected and trained on the prepared features to learn the mapping from molecular descriptors to fluorescent properties. Common supervised learning models for fluorescent property prediction include ensemble tree methods (such as random forests or gradient-boosting algorithms like XGBoost) and neural networks (including fully connected neural networks, convolutional neural networks, or graph neural networks). Simpler models like linear regression or decision trees might be chosen for small datasets, whereas deep neural architectures are favored for larger or more complex datasets. The choice often hinges on a trade-off between interpretability and predictive power. For instance, tree-based ensembles offer built-in feature importance metrics that help interpret which molecular features influence fluorescence, while neural networks can capture highly nonlinear relationships and often achieve higher accuracy at the cost of being “black boxes” with limited interpretability. In practice, the model architecture and type must be tailored to the nature of the available data (whether inputs are vectorized descriptors, spectra, molecular graphs, etc.) and the specific prediction task. It is also common to evaluate multiple candidate models or even use an ensemble of different learners to improve robustness and performance.

After training, rigorous model validation is performed to assess predictive performance and generalization ability. Typically, techniques like k-fold cross-validation or hold-out test sets are employed to ensure the model performs well on unseen data. Key regression metrics include the coefficient of determination (R^2^), mean absolute error (MAE), and root mean square error (RMSE), whereas classification tasks may use metrics such as accuracy, precision/recall, and ROC-AUC. High performance on training data must be balanced with checks against overfitting. For example, if data are limited, one might favor simpler models or apply regularization to prevent the model from simply memorizing the training set. By examining validation results, researchers can tune hyperparameters or revisit feature selection to further refine the model before deployment.

Finally, the validated model is deployed for property prediction or high-throughput screening of candidate materials. In fluorescent materials research, deployment could mean using the model to predict the emission wavelengths or quantum yields of new candidate molecules before synthesis, or scanning a virtual library of molecular structures to find promising luminophores (i.e., performing high-throughput virtual screening) [29,30,31]. In some cases, multi-task learning is employed, wherein a single model simultaneously predicts multiple related fluorescent properties (for example, both emission energy and quantum yield) to exploit correlations between those properties. For the model’s predictions to be practically useful, it is crucial that the model be not only accurate, but also generalizable—it should reliably predict properties for novel molecules or material compositions that were not explicitly represented in the training data. Throughout this workflow, iterative refinement is often necessary. As the scope of fluorescent materials under study expands, researchers periodically update the dataset with new experimental results, adjust feature sets, or retrain models to maintain and improve predictive performance. Increasingly, active learning strategies are integrated into this refinement loop, where the model actively suggests the most informative new experiments or data points to acquire, thereby efficiently improving itself with minimal experimental effort [30,32,33,34,35,36].

**Figure 2:**
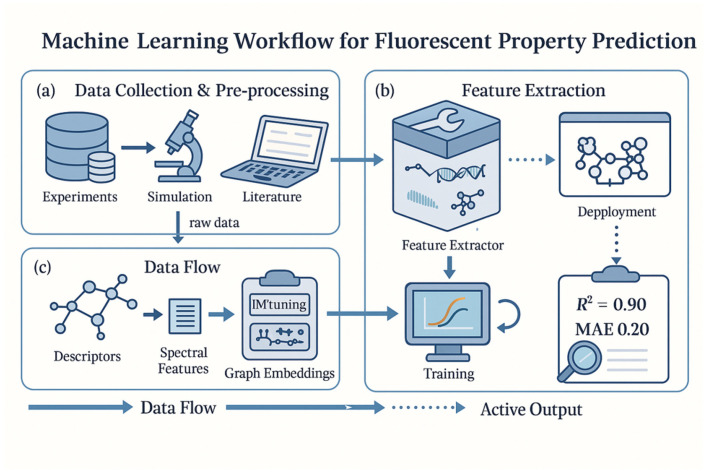
Schematic of a typical machine learning workflow for fluorescent property prediction. The diagram shows the sequential steps from (**a**) data collection and curation, through (**b**) feature extraction and (**c**) model development.

In summary, the ML workflow for fluorescent property prediction mirrors a standard data-driven modeling pipeline, but it must be executed with special attention to the nuances of photophysical data. Data quality and feature relevance set the upper limit on achievable model performance, while thoughtful model selection, hyperparameter tuning, and validation ensure the resulting model can be trusted to guide fluorescent material discovery. This foundational workflow underpins the more advanced machine learning strategies discussed in subsequent sections. As shown in Figure 2, the overall ML workflow spans data collection, feature engineering, model training, and iterative validation for fluorescence prediction.

### 2.2. Representative Learning Paradigms: Supervised, Self-Supervised, and Reinforcement Learning

Machine learning methods can be categorized by how they learn from data. The most widely used paradigm in materials informatics is supervised learning, in which models train on examples paired with known output labels (here, measured fluorescent properties). The goal of supervised learning is to learn a mapping from input features (e.g., molecular descriptors) to target outputs (e.g., emission wavelength) with high fidelity. Supervised learning has underpinned the majority of ML studies on fluorescent materials to date because typically many known compounds have experimental or simulated fluorescence data available as labels. For instance, a supervised regression model might be trained on a database of molecules with their measured absorption and emission peaks, then used to predict the photoluminescence wavelength of new molecules. Owing to its maturity and effectiveness, supervised learning remains the workhorse for most predictive tasks (such as quantitative structure–property relationship modeling) in this field. However, supervised approaches require substantial labeled datasets, and their performance can degrade when data are scarce or biased toward certain regions of chemical space.

**Figure 3:**
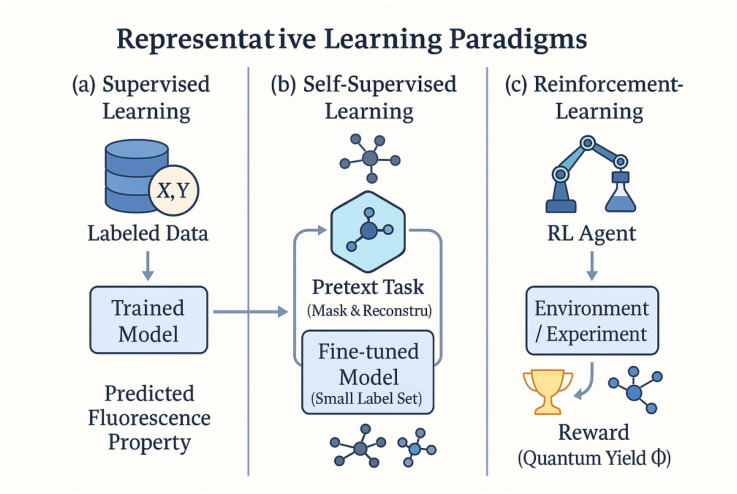
Illustration of representative learning paradigms in ML for fluorescent materials. (**a**) Supervised learning: The model trains on labeled examples of molecular structures and their known fluorescence properties. (**b**) Self-supervised learning: The model first learns from unlabeled data via a pretext task (such as predicting a masked part of a molecular structure) and is then fine-tuned on a smaller labeled dataset. (**c**) Reinforcement learning: An agent iteratively proposes new material candidates or experimental actions and receives reward feedback (e.g., a high predicted quantum yield), allowing for it to optimize fluorescent properties through trial-and-error exploration.

By contrast, self-supervised learning leverages unlabeled data to pre-train models via surrogate tasks, providing a way to exploit large pools of unannotated information (such as unmeasured molecular structures or unassigned spectra). In self-supervised schemes, the model learns intrinsic patterns or representations from the data itself—for example, by predicting missing parts of an input or by distinguishing altered inputs—and can then be fine-tuned on the actual prediction task using only a small amount of labeled data. This paradigm is valuable for fluorescent materials research because obtaining experimental fluorescence labels can be costly and time-consuming, whereas uncharacterized molecules or spectra are relatively plentiful. As shown in Figure 3, supervised learning relies on labeled structure–property pairs, while self-supervised learning leverages abundant unlabeled molecular or spectral data. One representative approach is to devise a “pretext” task on molecular structures, such as predicting masked fragments of a molecule or generating one data modality from another [37,38,39,40,41]. For example, Xie et al. introduced a chemistry-aware fragmentation strategy where a model is trained to reconstruct missing substructures of a molecule from the remaining parts [42]. This self-supervised pre-training approach (termed CAFE-MPP) forces the model to learn chemically meaningful features of molecules, which in turn improves its performance on downstream fluorescent property prediction tasks [42]. More generally, by pre-training on large unlabeled chemical libraries and then fine-tuning on smaller labeled fluorescent-material datasets, self-supervised learning can significantly boost predictive accuracy and robustness. It effectively acts as a form of knowledge transfer: the model extracts general chemical feature representations (for example, molecular motifs relevant to electronic transitions) from vast unlabeled data, which helps it predict fluorescence behavior even when only limited training examples are available for the target task. Self-supervised paradigms are still emerging in this field, but they hold great potential to alleviate data scarcity and to uncover nuanced structure–property relationships that might be missed by purely supervised models.

Another promising paradigm is reinforcement learning (RL), which is fundamentally different from both supervised and self-supervised approaches. In reinforcement learning, an agent learns to make a sequence of decisions through trial-and-error interactions with an environment, guided by a feedback reward signal rather than direct example labels. Instead of training on fixed input–output pairs, the RL agent actively explores different actions (for example, proposing a new molecular structure or adjusting a synthesis parameter) and receives higher rewards when those actions yield a desirable outcome (such as a high predicted quantum yield). This approach is well-suited for optimization and design problems in fluorescent materials science. For instance, researchers have applied RL to inverse molecular design of fluorophores, with the goal of discovering novel fluorescent molecules that have optimal properties beyond those present in the training set [43]. One notable strategy is to use RL to navigate chemical space by assembling a molecule step-by-step: the agent adds chemical fragments one at a time, treating each addition as an action, and it is rewarded for constructing molecules that meet target fluorescence criteria. Kim et al. demonstrated an RL-guided molecular generator capable of finding emissive compounds with extreme target properties that conventional models (which are bound by learning the training data distribution) failed to discover [44]. This result highlights RL’s strength in extrapolation—it can search beyond the domain of known examples by prioritizing high-reward (high-performance) candidates rather than strictly reproducing the patterns seen in the training data. Moreover, domain knowledge can be incorporated into RL by shaping the reward function (for example, penalizing structures that violate chemical stability rules), and RL naturally handles multi-step decision processes such as multi-stage syntheses or sequential screening experiments. In the context of fluorescent materials, one can envision RL-driven systems that suggest which molecule to synthesize next or how to tune processing conditions to maximize emission intensity. Early studies have even combined RL with generative models (for example, using an RL policy to guide a generative adversarial network in molecule creation) and reported promising success in creating unique candidate fluorophores [44,45]. Overall, reinforcement learning provides a powerful tool for active discovery and optimization in fluorescent material research, complementing passive predictive models with an ability to perform goal-directed searches through vast chemical and process parameter spaces.

### 2.3. Key Modeling Techniques: Attention, Multimodal Learning, Transfer, and Interpretability

To further improve the performance and usefulness of ML models for fluorescent materials, researchers have adopted several key modeling techniques in recent years. One such advancement is the incorporation of attention mechanisms, which allow for models to dynamically weight the importance of different parts of the input. Attention was originally popularized in natural language processing (e.g., the Transformer architecture) and has since been adapted to chemical representations and even spectral data. By learning where to “focus”, an attention-enabled model can highlight which atoms or bonds in a molecule are most influential for its fluorescence. In sequence-based models that operate on linear representations like SMILES strings, self-attention helps capture long-range dependencies—for instance, a distant substituent that affects a molecule’s emissive properties—more effectively than traditional recurrent neural networks. Similarly, in graph-based models, the graph attention network (GAT) variant extends a standard graph neural network by assigning learnable weights to neighboring nodes in a molecular graph. This means the model can pay greater attention to specific atomic interactions (for example, a particular conjugated bond or an electron-donating group) that strongly impact excited-state behavior, while ignoring less relevant parts of the structure. The incorporation of attention not only tends to improve prediction accuracy, but also provides a measure of interpretability: the learned attention weights can be visualized as an “importance map” over the molecule or input features. For example, an attentive model might automatically learn that the presence of a donor–acceptor pair in a TADF emitter deserves high attention due to its effect on the singlet–triplet energy gap, thereby implicitly identifying a key substructure. Indeed, one study on crystalline materials introduced a crystal GAT model that improved the prediction of new stable compounds by highlighting critical local chemical environments [46]. Overall, attention mechanisms make ML models for fluorescent materials more flexible in modeling complex relationships and more transparent in explaining which features drive their predictions.

Another important approach is multimodal learning, which involves integrating multiple forms of data or representations into a single predictive model. Fluorescent material properties are often governed by a combination of factors that can be captured in different data modalities. For instance, a molecule’s fluorescence quantum yield might depend on its molecular structure and on its excitation/emission spectrum or environment. Rather than building separate models for each factor, multimodal ML techniques aim to learn a joint representation from all available data types to better predict outcomes. In practice, this could mean combining chemical structural descriptors with optical spectra, images, or even textual information about experimental conditions in one unified framework. Such integration has proven powerful in recent studies—for example, the modeling of aggregation-induced emission (AIE) luminogens was significantly improved by using hybrid descriptors that include both molecular structural features and aggregate-state spectral features [47]. By training on these multimodal inputs, the model captures how structural factors and spectral behavior together influence emission efficiency. This strategy is especially valuable when single-modality data is insufficient to account for complex photophysical phenomena. We are also seeing “cross-modal” approaches, where a model might, say, translate an experimental absorption spectrum into a prediction of the most likely molecular structure or vice versa (akin to a multimodal translation task). Incorporating multiple data sources tends to make models more robust as well: a multimodal model is less likely to overfit to the quirks of one particular data type, and it can generalize better by finding consistent patterns that appear across different modalities. As public datasets for fluorescent materials continue to grow (including spectral libraries, molecular databases, etc.), multimodal learning is expected to become increasingly prominent, enabling richer predictive insights than any single data stream alone.

Transfer learning is another technique that has become crucial in this domain, helping to address the challenge of limited data for specific fluorescent material classes. In transfer learning, knowledge gained from one task or dataset is transferred to improve learning on a related second task or dataset. A common scenario in fluorescent materials research is that we have abundant data for one type of system or property, but far less for another—for example, thousands of measurements for quantum dots versus only a few dozen for a new class of organic dyes. Rather than training a model from scratch on the small dataset, one can first pre-train a model on the larger, related dataset and then fine-tune it on the target task with the smaller dataset. This approach has been shown to significantly boost performance in fluorescence property prediction. For instance, Jeong et al. demonstrated a deep learning model that was initially trained on a large corpus of optical spectra and then adapted via transfer learning to predict orbital energy levels (HOMO/LUMO) of organic fluorophores [48]. The transfer-learned model achieved substantially higher accuracy than a model trained directly on the small fluorophore dataset [48,49,50]. The success of transfer learning in such contexts stems from the fact that many photophysical patterns and mechanisms are shared across different material systems. A neural network that has already learned general spectral features or molecular feature patterns from one dataset (e.g., inorganic phosphors or quantum dots) can reuse those learned representations to more efficiently learn the behavior of another dataset (e.g., organic emitters). In essence, the model carries over a “head start” in recognizing which molecular characteristics correlate with fluorescence properties. Transfer learning is especially powerful when combined with careful fine-tuning, wherein the model’s parameters are adjusted gradually on the new data to avoid catastrophic forgetting of the earlier knowledge. This technique reduces the amount of experimental data required in the target domain and accelerates model development. It also opens up possibilities like cross-modal transfer—for example, using a model pre-trained on computationally generated spectra (from simulations) to help interpret experimental spectra—effectively transferring knowledge from a simulated domain to an experimental one. As the fluorescent materials field diversifies with new emitters and operating conditions, transfer learning provides a practical pathway to leverage data-rich domains and jump-start modeling in emerging areas.

Finally, ensuring model interpretability is a key concern, given that scientific understanding and trust are as important as raw predictive accuracy in materials research. Interpretability techniques aim to explain or rationalize the predictions of complex models, helping researchers gain insight into the structure–property relationships that the model has learned. There are several approaches to achieve this. One straightforward method is to use inherently interpretable models (such as decision trees or sparsified linear models) or to examine feature importance scores in ensemble methods. For example, random forest and XGBoost models can rank which input descriptors (e.g., molecular weight, band gap, conjugation length, etc.) most strongly influence the fluorescence output, yielding human-understandable insights (e.g., “conjugation length and donor strength were the top predictors of emission wavelength”). However, for more complex models like deep neural networks, post hoc interpretability tools are required. Techniques such as Shapley value analysis and saliency maps have recently been applied to chemical ML problems. For instance, a method called MolSHAP was developed to compute Shapley additive explanations at the level of molecular substructures, quantifying how much each functional group contributes to a model’s predicted property [51]. Using MolSHAP, a model predicting quantum yield might reveal that the presence of a heavy-atom substituent subtracts a certain amount from the predicted yield (due to enhanced intersystem crossing), whereas a rigid planar core adds to the predicted yield (by reducing non-radiative decay). Such explanations align well with chemical intuition, thereby validating the model’s reasoning or, conversely, highlighting when the model might be relying on spurious correlations. Another set of interpretability techniques involves leveraging the model’s internal attention weights (as mentioned above) or conducting counterfactual analyses—for example, seeing how small, targeted modifications to a molecule’s structure would affect the model’s prediction. Recent developments in explainable AI for chemistry have even begun integrating physical knowledge into the interpretability process: for example, explainable graph neural networks can be constrained to attend to chemically meaningful features like aromatic rings or charge-transfer pathways [52]. By making ML models more transparent, researchers can trust and act on their predictions more readily, which is crucial for adopting ML-guided design of new fluorescent materials. Moreover, improved interpretability often leads to new scientific hypotheses: if a model consistently points to a particular molecular motif as crucial for high brightness or stability, that insight can guide the rational design of next-generation luminophores and prompt targeted experiments to verify the model’s suggestions.

**Figure 4:**
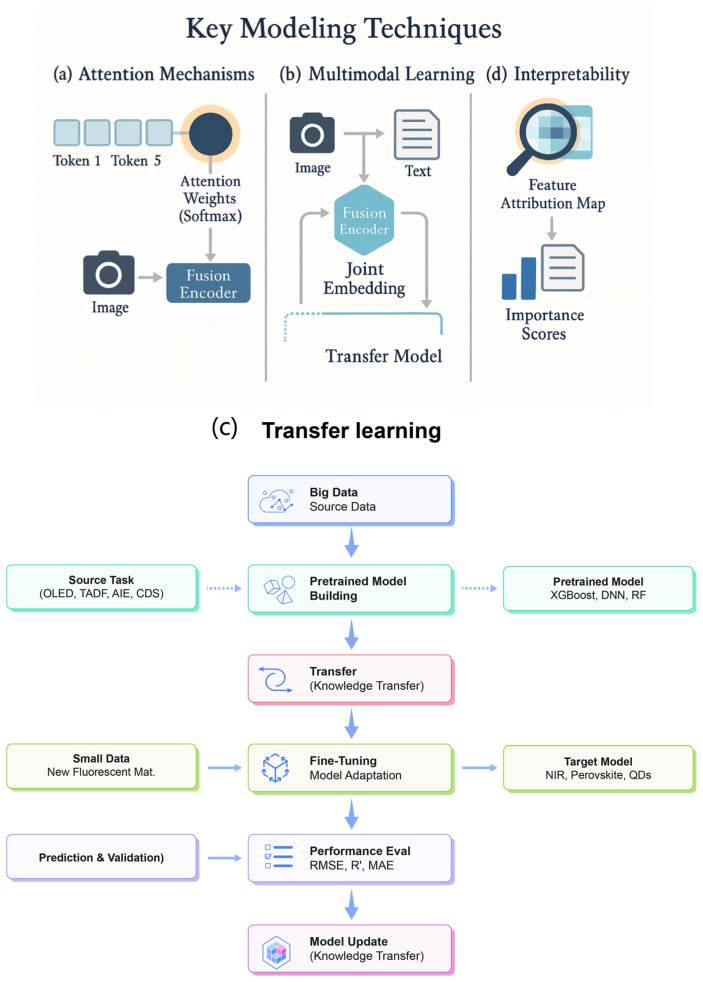
Key modeling techniques enhancing ML models for fluorescent materials. (**a**) Attention mechanisms: The model dynamically highlights influential parts of the input (e.g., certain atoms or bonds in a molecule are given higher weight, indicated by darker shading). (**b**) Multimodal learning: the model combines multiple data types (such as molecular structure and spectral data) to learn a joint representation for predicting fluorescence. (**c**) Transfer learning: Knowledge from a data-rich source task (gray, left) is transferred to improve performance on a related data-scarce target task (blue, right). (**d**) Interpretability: Tools like feature importance maps or Shapley values explain which molecular features or descriptors most affect the model’s predictions, aligning the model’s reasoning with chemical intuition.

In summary, attention mechanisms, multimodal data integration, transfer learning, and interpretability methods are all pivotal techniques that enhance the capabilities of ML models to not only predict fluorescent material properties more accurately, but also to do so in a manner that yields scientific insight and practical trust. Together, these techniques help bridge the gap between black-box predictions and actionable understanding, making data-driven models more useful and credible in real-world fluorescent materials research. It can be seen from Figure 4 that attention mechanisms, multimodal fusion, and physics-informed models enhance interpretability and robustness.

### 2.4. Emerging Architectures and Algorithms: GNNs, GATs, Diffusion Models, Meta-Learning, and Bayesian Optimization

As the field progresses, several advanced ML architectures and algorithms have emerged that show great promise for fluorescent materials research. One major development is the rise of graph neural networks (GNNs). Unlike traditional neural networks that require fixed-length vector features, GNNs operate directly on graph-structured data—in this case, molecular graphs or crystal lattices, where nodes represent atoms and edges represent bonds or interactions. GNN models (including message-passing neural networks) naturally capture the connectivity and local chemical environments of a molecule, making them ideal for predicting properties that fundamentally derive from molecular structure. In fluorescent materials, GNN-based models have been successfully used to predict outcomes like emission wavelength and stability by training on large datasets of molecular structures. These models often outperform traditional descriptor-based approaches because they do not rely on human-defined features; instead, the network learns its own relevant features during training (for example, automatically recognizing subgraph patterns that correspond to chromophores or quenchers). An important extension of the GNN is the graph attention network (GAT), which incorporates attention mechanisms into the graph model. GATs allow for the network to weight different bonds or neighboring atoms unequally, effectively learning which parts of the molecular graph are most important for the property of interest. This has both accuracy and interpretability benefits. For example, a GAT model might focus attention on the π-conjugated core of a molecule when predicting its fluorescence color, aligning with chemical intuition that the conjugated system dictates emission wavelength. Indeed, a study on crystalline materials introduced a crystal GAT model that improved the prediction of new stable compounds by highlighting critical local chemical environments [22]. For luminescent molecular crystals or metal–organic frameworks, such graph-attention models could similarly identify key structural motifs (like specific ligand–metal interactions or packing features) that influence photoluminescence. As research pushes into more complex material systems and larger molecular datasets, GNNs and GATs provide scalable and insightful modeling tools that directly leverage the structural formula or crystallographic data of fluorescent materials.

Another cutting-edge development is the advent of diffusion models for generative tasks. Diffusion models are a class of deep generative networks that have recently proven highly effective in domains like image synthesis, and they are now being adapted for molecule generation and materials design. The basic idea is that a diffusion model learns to invert a gradual noising process: it starts from a random noise input and iteratively “denoises” it to produce a structured output such as a molecular graph. In the context of fluorescent materials, diffusion models enable a new approach to de novo molecular design—one can generate novel molecular structures that have desired fluorescent properties by appropriately guiding the diffusion process. A powerful example is the Guider of Autoencoding Diffusion for Individuals (GaUDI) framework. In a recent study, GaUDI was trained on a large dataset of polycyclic aromatic compounds (many of which are classic organic fluorophores) and then used to generate new molecular structures optimized for multiple target properties (such as specific HOMO–LUMO energy gaps and thermal stabilities) [53]. By incorporating a property prediction module into the generative loop, the diffusion model was biased toward producing candidates with the desired photophysical characteristics. Remarkably, this diffusion-based approach was able to propose chemically valid, diverse molecules that went beyond the training set distribution, achieving nearly 100% validity and uniqueness in the generated candidates [53]. This result indicates a strong potential for discovering truly novel emitters that conventional generative techniques or human intuition might miss.

Compared to earlier generative techniques like variational autoencoders (VAEs) or generative adversarial networks (GANs), diffusion models tend to provide more stable training and higher-quality outputs, and they can be more readily conditioned on complex design objectives. For fluorescent materials design, one can imagine using diffusion models to simultaneously optimize a molecule’s structure for a set of criteria—for example, maximizing quantum yield and photostability while also hitting a target emission color—by formulating those objectives into the diffusion model’s guidance or reward function. This capability aligns well with the multi-objective nature of materials discovery. As these models mature, diffusion-based generative design could become a mainstream approach to propose new fluorescent compounds (or even device structures) that meet stringent performance requirements before any lab synthesis is attempted. This generative capability essentially enables an inverse design strategy: rather than predicting properties from a given structure (the traditional forward modeling approach), the ML model works backward from desired target properties to suggest new molecular structures that are likely to exhibit those properties [53,54].

In parallel, the concept of meta-learning (or “learning to learn”) has gained traction as a way to tackle the small-data problem that often plagues specialized fluorescent material studies. Meta-learning algorithms improve their learning efficiency by leveraging experience from multiple learning tasks. In practical terms, a meta-learning approach might train a model on a variety of related tasks (each with limited data) to develop an adaptable model that can quickly fine-tune to a new task with only a few examples. This is highly relevant in cases where for example, a researcher has several small datasets—perhaps fluorescence data under different environmental conditions or for different families of luminophores—and wants a model that can handle a brand-new case with minimal additional data. One popular form of meta-learning is few-shot learning, which aims to achieve reasonable performance given just a handful of training samples by relying on knowledge distilled from previous tasks. In the fluorescent materials domain, few-shot meta-learning could manifest as follows: during training, the model is exposed to many different fluorescence-prediction tasks (e.g., predicting emission peaks for coumarins, for BODIPY dyes, for quantum dots, etc.) and through this process it learns a universal representation of “what generally controls fluorescence”. When faced with a new task—say, predicting properties for a novel class of lanthanide-doped phosphors with only a very few measured data points—the meta-trained model can quickly adapt to this task, often requiring only a handful of additional examples to reach good accuracy.

Recent studies have started to demonstrate the power of meta-learning in materials science. For instance, Allen et al. showed that a meta-learning approach could fit interatomic potential models across multiple levels of theory, resulting in models that adapted to new atomic systems with significantly reduced error using very little training data [55]. Analogously, for fluorescence problems, a meta-learned model could be quickly calibrated to a new measurement setup or a new molecular scaffold with minimal data, leveraging prior knowledge gained from related fluorescence datasets. Another benefit of meta-learning is its ability to handle noisy or inconsistent data. Meta-learning algorithms have shown resilience in scenarios with high label noise or varying data quality, which is useful since experimental fluorescence data often come from different labs or methods and can vary in reliability [56,57]. While still a developing area, meta-learning holds promise for producing more generalizable and adaptable ML models in fluorescent materials research. It effectively serves as a route toward “foundation models” for this field—large-scale models that capture general patterns of fluorescence behavior and can then be specialized to myriad specific prediction tasks with minimal effort.

Beyond model architectures and learning paradigms, advanced approaches in closed-loop optimization are transforming how researchers search for optimal fluorescent materials or experimental conditions. One such approach is Bayesian Optimization (BO), which has emerged as a powerful algorithm for guiding both experimental design and hyperparameter tuning in materials science. BO is a sequential optimization strategy particularly well-suited for expensive-to-evaluate problems, such as actually synthesizing a new material or running a costly high-accuracy simulation. It works by building a probabilistic surrogate model (often a Gaussian process) of the target objective function and then selecting the next query point by balancing exploration and exploitation (typically via an acquisition function). In fluorescent materials discovery, for example, one might use BO to suggest the next material composition or molecular structure to test in order to maximize a target metric like fluorescence quantum yield or color purity. The BO algorithm uses past observations (e.g., which materials have been tried and their measured properties) to model the objective surface and intelligently propose new candidates that are predicted either to be very high-performing or to significantly reduce uncertainty. One notable advantage of BO is its principled way of incorporating uncertainty: it naturally gravitates towards experiments that are predicted to be highly promising (high reward) or those that would be very informative (high uncertainty and thus high potential information gain). This is ideal in domains where experiments are costly or time-consuming, and one cannot brute-force through thousands of candidates.

Researchers have applied BO in fluorescent materials research to efficiently navigate large search spaces with minimal experiments. For instance, BO has been used to optimize the synthesis conditions for two-dimensional material phosphors, achieving improvements in photoluminescence by efficiently searching the space of growth parameters [58]. Similarly, in an autonomous laboratory setting, BO has helped identify optimal compositional blends of OLED emitters, discovering formulations that yield long device lifetimes and high efficiency in far fewer experimental iterations than a grid or random search would require. Beyond purely data-driven optimization, physics-guided variants of BO further enhance its power by integrating known physical relationships or constraints into the model. For example, a physics-informed BO approach was demonstrated for materials discovery where the model was constrained by Vegard’s law (a rule describing how lattice constants vary with composition), which enabled more reliable extrapolation beyond measured composition ranges [59]. By embedding such prior knowledge, BO can avoid suggesting implausible or unphysical candidates and focus on the most promising regions of material space that respect known scientific rules.

**Figure 5:**
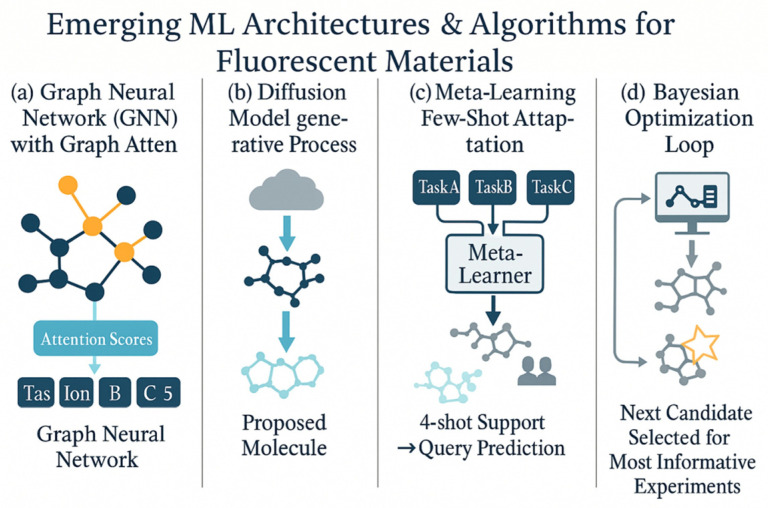
Emerging ML architectures and algorithms for fluorescent materials. (**a**) Left: A graph neural network (GNN) operating on a molecular graph, with a graph attention mechanism (highlighted in orange) indicating the most important bonds or atoms for predicting a fluorescence property. (**b**) Center: A diffusion model’s generative process, transforming random noise into a structured molecule through iterative refinement, guided by a property evaluator to satisfy target emission criteria (an inverse design approach to propose new emitters). (**c**) Top right: A meta-learning paradigm where a model trained on multiple related tasks can quickly adapt to a new prediction task with only a few training examples (few-shot learning). (**d**) Bottom right: A Bayesian optimization loop in which a surrogate model predicts performance and an acquisition function selects the next experimental candidate (orange star) to test, efficiently searching for high-performing materials (often combined with active learning to choose the most informative experiments).

In summary, Bayesian optimization offers a data-efficient strategy to optimize fluorescent materials and their processing, effectively closing the loop by connecting model predictions with experimental decision-making. It is often used in tandem with complementary techniques like reinforcement learning or active learning to guide the selection of subsequent experiments [58]. For example, an active learning approach might analyze a model’s uncertainty to choose the next material candidate whose experimental evaluation would maximally improve the model [60,61]. Together, these emerging architectures (GNNs, GATs, diffusion models) and algorithms (meta-learning, BO, and related strategies) are rapidly expanding the researcher’s toolkit, enabling faster and smarter exploration of the vast design space of fluorescent materials and facilitating the inverse design of new luminophores with desired properties. As illustrated in Figure 5, GNNs, diffusion models, and generative frameworks empower inverse design and discovery of novel emitters.

### 2.5. Data Efficiency Strategies: Augmentation, Weak Supervision, and Physics-Guided Learning

One of the central challenges in applying ML to fluorescent materials is the limited quantity and diversity of high-quality data available for training models. To address this, a number of data-centric strategies have been developed to improve model generalization without requiring massive new experimental datasets. Data augmentation is a first-line approach, wherein the training dataset is artificially expanded by transforming existing data or generating synthetic data that preserves the essential information. In the context of image-based ML, augmentation techniques (like rotating or cropping images) are commonly used; analogously, for molecular and spectral data, one can create variations that maintain the underlying labels. For example, a popular technique for molecules is SMILES augmentation. A single molecule can be represented by many different, but equivalent, SMILES strings (due to the arbitrary ordering of atoms in the notation). By generating multiple randomized SMILES permutations for each molecule, one can effectively multiply the dataset size without collecting new experimental data. This practice has been shown to improve model robustness, as the ML model becomes less sensitive to any particular token ordering and instead learns more general chemical features [62]. Another advanced augmentation method for molecular strings uses the SELFIES (Self-Referencing Embedded Strings) encoding, an alternate text representation that guarantees a valid molecule for any sequence of symbols. Augmenting training data with slight random mutations in the SELFIES representation can introduce novel but still chemically valid samples, which is particularly helpful for preventing overfitting when the original dataset is very small [63,64].

Beyond molecular structure representations, spectral augmentation is also valuable when dealing with optical data. Techniques such as adding random noise to spectra, shifting peak positions, or even mixing parts of different spectra have been employed to simulate measurement variability and broaden the coverage of the training data. For instance, creating several noisy versions of an experimental fluorescence spectrum can teach a model to ignore minor instrumental fluctuations and focus on the true spectral features, thereby improving its robustness in real-world prediction scenarios. Studies have reported that models trained with augmented spectral and structural data yield higher accuracy and better generalization, especially for tasks like predicting photoluminescence quantum yields or near-infrared emission peaks where the initial training data might be very limited [65]. In summary, data augmentation leverages known invariances and noise patterns in the data to supply the model with a richer variety of training examples. This ultimately reduces overfitting and makes the model more trustworthy when predicting fluorescence properties for new materials.

In cases where obtaining perfectly accurate ground-truth labels is particularly difficult, weak supervision offers a way to utilize imperfect or proxy data to train ML models. Weak supervision is an umbrella term for methods that learn from noisy, partially correct, or indirectly obtained labels instead of relying solely on small amounts of pristine data. In the context of fluorescent materials, this could mean using theoretical or semi-empirical calculations as provisional labels for training, or mining the scientific literature for reported fluorescence values that come with some uncertainty. For example, one strategy is to perform high-throughput virtual screening by using computational chemistry to generate labels: one could calculate thousands of approximate excitation energies with DFT as substitutes for experimental absorption maxima [66]. While individual DFT-calculated values might have systematic errors, collectively they can provide a useful learning signal across a much larger number of compounds than could be measured experimentally. Another form of weak supervision is leveraging distant supervision from published text: algorithms can scan journal papers or databases to extract mentions of molecules and their fluorescence properties, essentially constructing a rough dataset from the literature (with the understanding that these extracted labels may not be perfectly reliable) [67]. These noisy or proxy labels can then be used to train a model, ideally with techniques that account for label uncertainty. For instance, the model might be trained to predict a distribution of possible property values rather than a single point estimate, reflecting the uncertainty in the training labels.

Research in related fields has shown that models can be trained on large, weakly labeled datasets to achieve performance close to that of models trained on a smaller set of clean, high-accuracy labels [68]. A key trick is often to combine multiple weak signals so that they can compensate for one another’s errors. For example, one might combine a simple heuristic based on chemical intuition with a rough predictor; the model is then encouraged to learn the underlying fluorescence property that is consistent with both sources. Semi-supervised learning is another approach in this vein: one can train an initial model on the small set of labeled data, then use that model to predict labels for a larger pool of unlabeled data, and iteratively retrain. This process (sometimes called self-training) allows for the model’s own predictions on unlabeled fluorescent materials to become additional training data, gradually expanding the effective dataset. Overall, the effect of weak supervision methods is to broaden the training pool by accepting that not all labels are fully accurate. In fluorescent materials discovery, where experiments can be slow and expensive, these methods allow for researchers to incorporate cheaper information sources (computational simulations, expert chemical knowledge, existing databases with approximate values) to mitigate data scarcity. The outcome is often a model that, while not as precise as one trained on an enormous perfectly labeled dataset, still captures the general trends and can effectively rank or filter candidate materials for further investigation.

A complementary strategy to purely data-driven approaches is physics-guided learning, which integrates domain knowledge from physics or chemistry into the ML model to make more efficient use of limited data. Instead of treating the model as a completely black-box predictor, physics-guided methods introduce known scientific constraints or biases into the model’s structure or training objective so that it respects established principles of fluorescence. This can significantly reduce the amount of data needed, because the model does not have to re-discover fundamental relationships that are already well-known to human experts—those relationships are built in from the start. One way to implement this is by adding physical constraint terms to the model’s loss function during training. For example, one could enforce that a model’s predicted emission energy and absorption energy for a molecule never violate the typical range of Stokes shifts observed in practice, or ensure that the model’s predictions obey known selection rules (e.g., assigning lower scores to forbidden transitions that should have negligible oscillator strength). Another approach is to incorporate physics awareness into the feature set or model architecture: for instance, providing the model with physically meaningful input features (such as an estimate of an excited-state lifetime or a quantum yield computed from a simple theoretical formula) can guide it toward the correct relationships more quickly. A clear illustration of physics-guided ML in materials science is the development of physics-informed neural networks for material stability prediction, where embedding thermodynamic constraints (like phase stability conditions or defect formation energy limits) into the model prevented nonsensical outputs and even enabled the model to extrapolate sensibly into regimes with sparse data [68].

**Figure 6:**
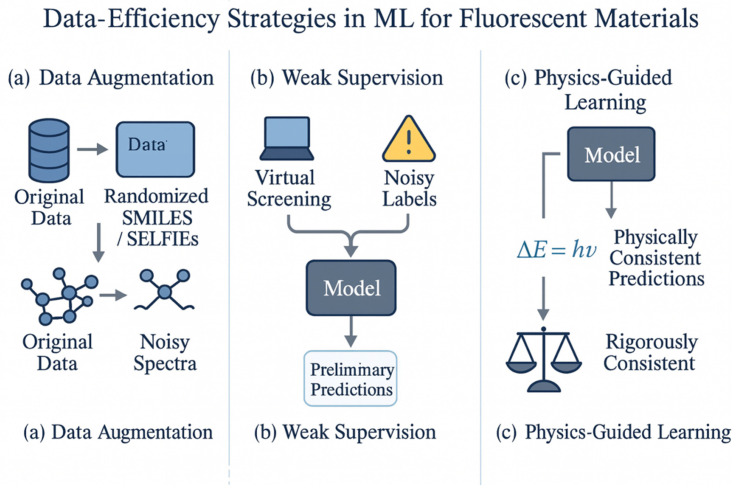
Data-efficiency strategies in ML for fluorescent materials. (**a**) Data augmentation: Expanding a limited dataset by creating modified versions of data points (e.g., multiple equivalent SMILES strings for one molecule, or synthetic noise added to spectra) to improve model generalization. (**b**) Weak supervision: Leveraging imperfect proxy labels from high-throughput virtual screening simulations or from mining literature reports when reliable experimental data are scarce. (**c**) Physics-guided learning: Incorporating known physical laws or constraints (such as energy conservation or empirical photophysical relationships) into model training so that predictions adhere to established scientific principles, reducing the amount of data required.

In fluorescent materials research, investigators have shown the value of physics-guided models for improving predictions under data-scarce conditions. For example, Chen et al. demonstrated that incorporating known radiative and non-radiative decay rate formulas into an ML model for fluorescence quantum yield led to markedly improved accuracy on novel compounds, even when training data were limited. (Notably, the physics-based constraints ensured the model would never predict a physically impossible quantum yield above 100% or below 0%, since those limits were hard-coded into the model’s allowable outputs.) Another example is the use of hybrid modeling, where a few steps of a physics-based simulation (such as a short molecular dynamics run to assess aggregation behavior) are performed for each candidate and the resulting physical insights (e.g., a metric of aggregation propensity) are fed into the ML predictor. In this way, the ML model benefits from physics-based hints about the system. We also see physics guidance being applied in the context of Bayesian optimization and active learning: for instance, incorporating Vegard’s law as a constraint when searching a compositional space of mixed crystals ensured that the ML-guided search only proposed candidates following known composition–property trends [68,69]. The net effect of physics-guided learning is a model that is more data-efficient and often more interpretable. By aligning the ML model with known scientific truths (for example, penalizing it if it violates energy conservation or known monotonic relationships), we effectively narrow the hypothesis space that the model must explore. This means fewer experimental data points are needed for the model to converge on a realistic solution that fits the observations. In fluorescent materials research, where we often juggle sparse data and complex phenomena, physics-guided ML serves as a vital bridge between the predictive power of data-driven algorithms and the reliability of established photophysical theory. It helps ensure that our models not only fit the data we have, but also make sense in light of decades of accumulated knowledge in fluorescence science—a crucial factor for the acceptance and success of ML-guided approaches in fluorescent material innovation. It can be seen from Figure 6 that strategies such as transfer learning, data augmentation, and weak supervision effectively mitigate data scarcity.

In conclusion, data-efficiency strategies such as augmentation, weak (or semi-) supervision, and physics-guided learning are enabling machine learning models to thrive even in the data-constrained scenarios typical of fluorescent materials research. These approaches extend and enrich the available information, mitigate overfitting, and enforce scientific consistency, thereby significantly enhancing the reliability and applicability of ML predictions for luminescent materials. Through the smart use of such strategies, researchers can extract maximal value from every experimental data point and confidently apply ML models to discover and optimize the next generation of fluorescent materials.

## 3. Machine Learning for Fluorescent Materials Across Systems

### 3.1. AIE Luminogens

Aggregation-induced emission (AIE) luminogens have emerged as a pivotal class of fluorescent materials due to their unique restriction of intramolecular motion (RIM) mechanism, which enables strong solid-state emission and has broad applications in bioimaging, sensing, and optoelectronics [70]. The application of machine learning (ML) in this field not only accelerates the discovery of novel AIE-active molecules, but also provides mechanistic insights into emission processes that are challenging to capture by conventional trial-and-error or quantum chemical approaches.

**Figure 7:**
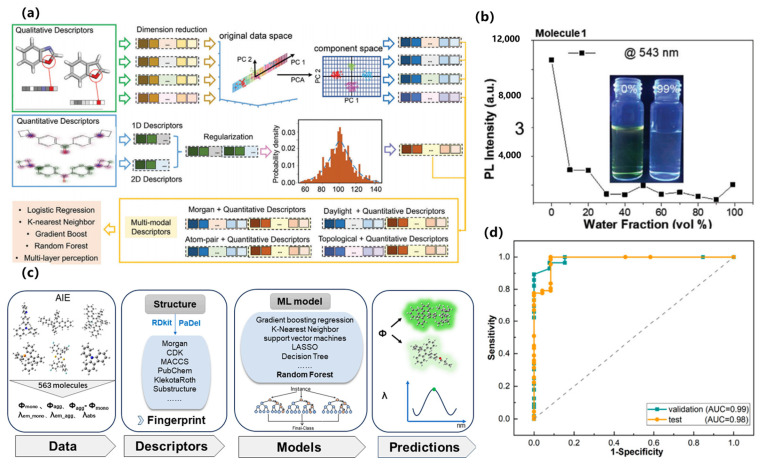
(**a**) Schematic illustration of multi-modal AIE descriptors. (**b**) Plots of the maximum photoluminescence (PL) intensity and the inset photographs of the compounds in 0 and 99 vol% water under UV light (365 nm) illumination. Reproduced from Ref. [71] with permission from the Wiley-VCH GmbH. (**c**) Workflow of machine learning (ML) approach in predicting the luminescence properties (quantum yield Φ and wavelength λ) of luminogens with aggregation-induced emission property (AIEgens) in the monomeric/aggregated states. (**d**) ROC curves of validation set predicted in ML training process and test set predicted with the optimal ML trained models for Φagg.

In terms of key modeling techniques, supervised learning and attention-based models have been widely applied to predict quantum yields, emission wavelengths, and mechanistic features of AIE luminogens. For example, Qiu et al. developed one of the earliest QM-ML hybrid approaches to distinguish AIE from aggregation-caused quenching (ACQ) molecules, successfully identifying RIM-related descriptors as critical predictive features [70]. Xu et al. subsequently introduced ML-assisted modeling to predict molecular optical properties upon aggregation, combining experimental and DFT-derived descriptors to enhance predictive accuracy [71], as shown in Figure 7a,b. More recently, Bi et al. employed advanced regression techniques to quantitatively predict both quantum yields and emission wavelengths, offering systematic guidelines for molecular design, as shown in Figure 7c,d [72].

With respect to emerging architectures, novel ML paradigms such as graph neural networks (GNNs) and photodynamics-informed frameworks have been adopted to address AIE complexity. Wang et al. applied ML to photodynamics simulations, uncovering non-radiative pathways blocked by aggregation that drive high luminescence, providing an interpretable link between structure and emission performance [73]. Peng et al. reported a ground-state descriptor–based virtual screening framework that leveraged interpretable ML to identify mechanofluorochromic AIE molecules, demonstrating the power of descriptor engineering in high-throughput discovery [74]. In addition, Zhang et al. extended ML models to AIE-active metal–organic frameworks (MOFs), revealing how ligand-level features control ensemble emission [75].

Regarding data efficiency strategies, multiple approaches have been explored to overcome the challenge of limited labeled AIE datasets. Zhao et al. demonstrated a weak-supervision strategy by integrating literature-mined data with curated experimental datasets to develop robust ML predictors for organic AIE materials [76]. Dave et al. highlighted multimodal learning by combining synthetic design features, photophysical measurements, and biological performance metrics to guide AIE molecular discovery in biomedical contexts [77]. Zhang et al. further showed that ML-assisted screening can significantly reduce the experimental burden by prioritizing candidates with strong fluorescence properties from large molecular pools [78]. Taken together, machine learning has enabled AIE research to transition from empirical optimization toward knowledge-driven discovery. The integration of supervised prediction, graph-based architectures, and data-efficient learning strategies has not only accelerated screening, but also provided mechanistic interpretability, underscoring its role as a cornerstone for the rational design of next-generation AIE luminogens.

### 3.2. Thermally Activated Delayed Fluorescence (TADF) Emitters

Thermally activated delayed fluorescence (TADF) materials have attracted extensive attention as next-generation emitters for organic light-emitting diodes (OLEDs), owing to their capability of harvesting both singlet and triplet excitons through reverse intersystem crossing (RISC), thereby achieving near-unity internal quantum efficiency [79,80]. The design of efficient TADF molecules, however, involves balancing frontier orbital separation, singlet–triplet energy gaps (ΔE_ST), and charge-transfer character, which renders conventional quantum chemical screening both computationally intensive and limited in scope. Machine learning (ML) has thus emerged as a transformative approach to accelerate the rational design and optimization of TADF emitters [81,82].

**Figure 8:**
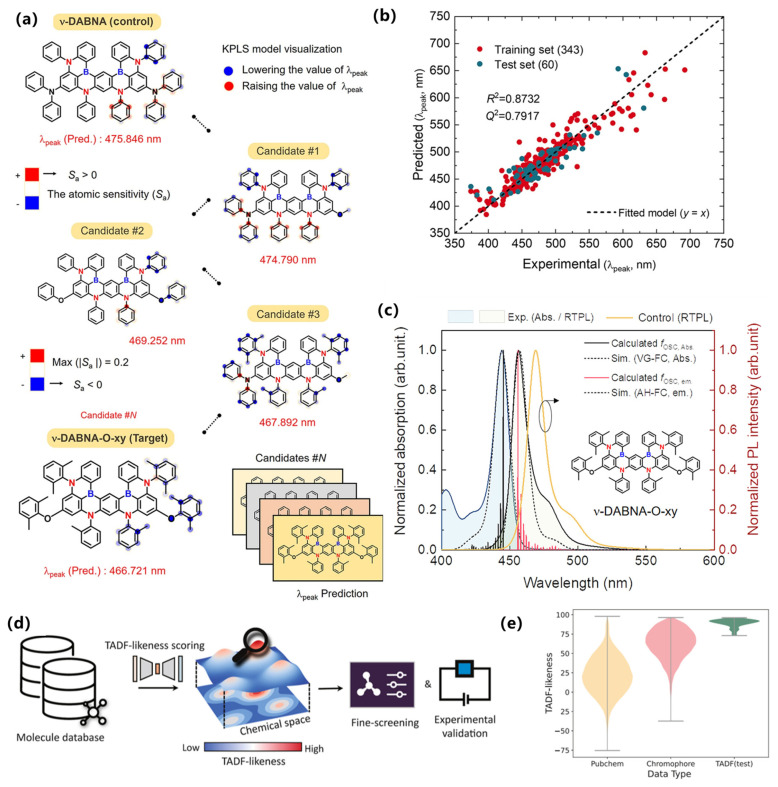
(**a**) Interpretable model visualization from radial-based KPLS regression model for λ peak prediction. (**b**) Scatter plot from the continuous model result, showing the training/test set of λ peak predictions. Reproduced from Ref. [84] with permission from the American Association for the Advancement of Science. (**c**) Absorption spectrum of ν-DABNA-O-xy and fluorescence spectra for the target and control molecules in toluene (concentration, 0.05 mM). (**d**) Overview of using the TADF-likeness score as a prefilter for high-throughput virtual screening. (**e**) The violin plots of TADF-likeness scores on various datasets. Reproduced from Ref. [87] with permission from American Chemical Society.

In terms of key modeling techniques, early ML efforts have largely employed supervised regression or classification using handcrafted features in TADF materials. For example, Shi et al. (2022) compiled a ~300-point database of TADF OLEDs with descriptors including photoluminescence quantum yield (PLQY), singlet–triplet gap (ΔE_ST), emission wavelength, host polarity, etc., and applied multiple algorithms (linear regression, random forest, neural nets, XGBoost) to predict external quantum efficiency (EQE) [83]. They found PLQY, emission wavelength and ΔE_ST to be the most important features governing EQE, as revealed by feature-importance analysis (e.g., RF/XGBoost). Similarly, Bu and Peng (2023) built an ML–QM high-throughput screening workflow for TADF (combining DFT calculations with an ML model) to flag promising emitters [29]. Complementing these approaches, an ML-QSPR workflow targeting multiresonant deep-blue systems nominated ν-DABNA-O-xy; subsequent synthesis and device testing confirmed narrowband emission and high efficiency, validating the in silico selection, as shown in Figure 8a–c [84].

Beyond mere prediction, ML has successfully guided the experimental discovery of novel TADF emitters. For instance, Bu and Peng employed an ML-assisted high-throughput virtual screening to identify 384 promising candidates from over 44,000 molecules, with subsequent quantum chemical validation confirming excellent TADF properties [85]. Remarkably, this approach was extended to full experimental synthesis and characterization by Shi et al., who developed an integrated ML-designed TADF molecule that was successfully synthesized and exhibited high performance in devices [86]. These cases exemplify a complete ML-guided pipeline, from in silico prediction to experimental validation, dramatically accelerating the discovery cycle. In parallel, a deep-learning chemical-similarity metric (“TADF-likeness”) has been introduced to pre-filter very large libraries and enrich downstream QSAR modeling, improving virtual-screening hit rates, as shown in Figure 8d,e [87]. Likewise, Bu and Peng (2023) used ML-assisted virtual screening to rapidly rank candidate TADF emitters, accelerating discovery by focusing on a narrowed chemical space [29]. Although graph-based GNNs or generative models have seen use in related luminescent materials, in TADF the literature remains dominated by tree-based regressors and neural networks. Multi-task learning (e.g., jointly predicting lifetime and efficiency) or Bayesian optimization could be applied to TADF design in future work.

Data-efficiency strategies have been less explored for TADF to date. The ML models above typically rely on published datasets and DFT-computed features, but only a few hundred data points exist. For example, Shi et al. note that experimental TADF data are sparse and non-IID, so they employ regularized models (ridge, Lasso) to mitigate overfitting [88]. In principle, active learning or physics-informed constraints (e.g., embedding rate equations for RISC) could reduce data needs, as has been performed in other luminescent systems, although specific TADF cases are not yet reported. Overall, ML-guided TADF design has shown promise in targeting key photophysical descriptors, but more sophisticated data-efficient schemes (active learning, multi-fidelity ML) remain a future opportunity.

### 3.3. Rare Earth–Doped Inorganic Phosphors (e.g., Ce/Eu^3+^)

Rare earth–doped inorganic phosphors, particularly those activated by Ce^3+^ and Eu^3+^ ions, are indispensable in solid-state lighting and display applications due to their high quantum efficiency, spectral tunability, and chemical stability [89]. However, optimizing luminescent properties such as emission wavelength, energy transfer efficiency, and thermal quenching typically requires laborious experimental synthesis and characterization. Machine learning (ML) methods have thus been introduced to accelerate the discovery and rational design of high-performance phosphors by efficiently mapping the complex relationships between host lattices, dopant environments, and photophysical outcomes.

**Figure 9:**
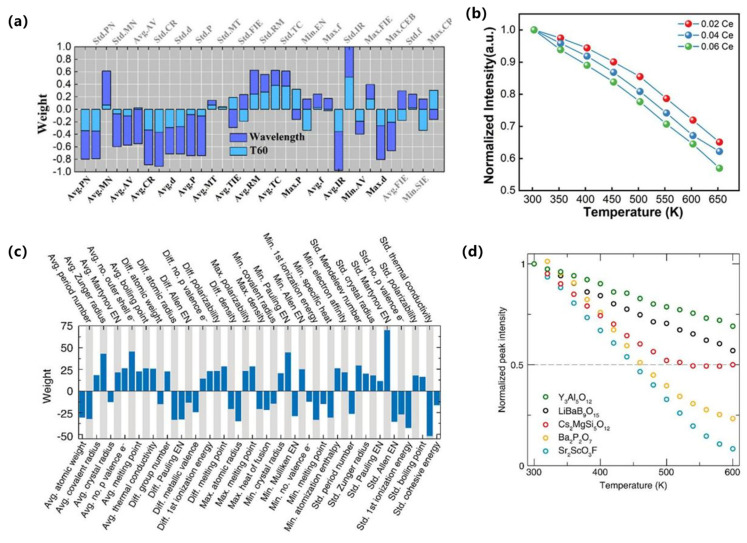
(**a**) Correlation comparison between the highly correlated features and two targets. (**b**) Temperature-dependent total emission intensity of Lu_1.5_Sr_1.5_A_l3.5_Si_1.5_O_12_:Ce from 303 to 653 K. Reproduced from Ref. [93] with permission from American Chemical Society. (**c**) Absorption spectrum of ν-DABNA-O-xy and fluorescence spectra for the target and control molecules in toluene (concentration, 0.05 mM). (**d**) Temperature-dependent total emission intensity of different materials. (Eu^3+^-Substituted). Reproduced from Ref. [94] with permission from American Chemical Society.

From the perspective of supervised learning approaches, regression and classification models have been widely employed to predict luminescence intensity, color coordinates, and thermal stability. For example, Park et al. reported a data-driven platform for Eu^2+^ phosphors that predicts band gap, excitation, and emission energies from 29 host descriptors, with ridge/lasso outperforming unregularized regressors and shallow ANNs on modest data [90]. Otsuka et al. reported a model for Eu^3+^-perovskites that predicts the intensity ratio Λ = I(^5D_0_→^7F_2_)/I(^5D_0_→^7F_1_) from chemical/structural features to control hue via site asymmetry (Otsuka, 2024) [91]. For Ce^3+^ systems, Zhuo et al. reported that crystal-field–motivated descriptors (ionic radii/charges) accurately estimate the 5d-level centroid shift ε_c across hosts (Zhuo, 2020) [92]. In addition, Zhuo et al. quantified feature–target correlations and temperature-resilience benchmarks in Ce^3+^-garnets, providing a quantitative baseline for ML-guided stability optimization, as shown in Figure 9a,b [93].

With regard to emerging architectures, While deep or graph-based models remain less common than classical regressors in Ce/Eu phosphors, Otsuka et al. further reported spectrum-level agreement between predicted and measured profiles, evidencing end-to-end fidelity from descriptors to optical response (Otsuka, 2024) [91]. Complementarily, Ding et al. reported hierarchical clustering that delineates host families conducive to targeted emission profiles, offering a data-driven map for composition-space navigation, as shown in Figure 9c,d [94]. These advances complement supervised baselines by adding spectral validation and unsupervised structure discovery, respectively.

To address data scarcity and generalization challenges, non-IID datasets, Park et al. reported explicit regularization (ridge/lasso) to stabilize inference and extract robust trends (Park, 2021) [90]. Koyama et al. reported a practical ML–experiment loop in which a classifier first predicts Eu oxidation state (+2 vs. +3) prior to targeted synthesis, yielding twelve Eu^2+^ phosphors from thirteen trials (Koyama, 2024) [95]. Zhuo et al. reported physics-guided feature construction that embeds crystal-field intuition into Ce^3+^ predictors, improving interpretability and sample efficiency (Zhuo, 2020) [92]. Zhang et al. used GPT-4 to extract Eu^2+^ phosphor data from 274 papers, training a CGCNN model (test R^2^ = 0.77) to predict emission wavelengths. This showcases a powerful closed-loop where ML builds the dataset for its own discovery, drastically reducing manual effort [96]. Collectively, these strategies move the field beyond black-box regression toward interpretable, feedback-rich pipelines that shorten the path from compositional hypotheses to verified Ce/Eu emitters, as shown in Figure 9a–d [94].

### 3.4. Transition-Metal–Doped (e.g., Mn^4+^/Cr^3+^/Fe^3+^)

Transition-metal–doped phosphors, typically activated by Mn^4+^, Cr^3+^, or Fe^3+^ ions, have attracted significant attention for applications in wide-color-gamut displays, plant lighting, and near-infrared bioimaging [97,98,99]. Compared with rare-earth activators, transition metals exhibit broad-band d–d or charge-transfer transitions that are highly sensitive to local coordination environments and lattice distortions. This complexity, while beneficial for tunable emission, presents major challenges in rational design and performance optimization. Machine learning (ML) has therefore emerged as a powerful approach to unraveling structure–property relationships and guiding targeted synthesis of high-efficiency transition-metal phosphors.

**Figure 10:**
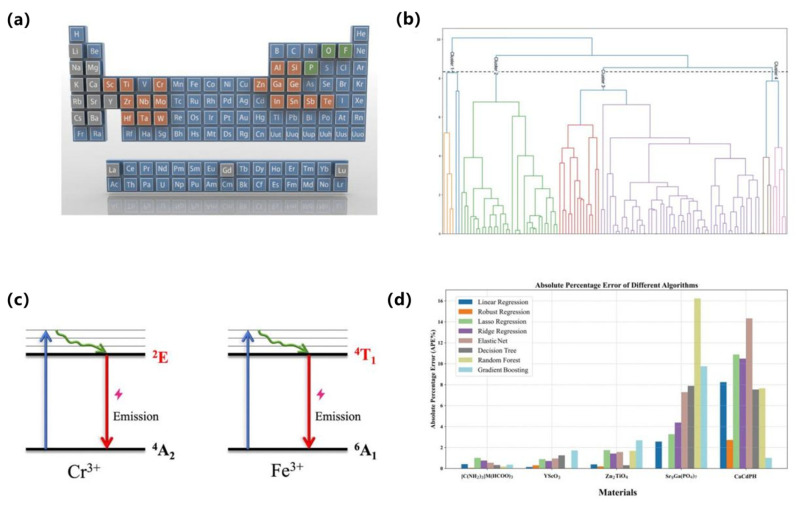
(**a**) The elements involved in Mn-^4+^ substituted phosphor hosts. The five positions are represented by three colors, respectively. The hosts involved in the dataset were restricted to the highlighted elements. (**b**) Hierarchical cluster analysis dendrogram identifying four main clusters of phosphors based on few easily obtained descriptors (Mn^4+^). Reproduced from Ref. [100] with permission from the Royal Society of Chemistry. (**c**) Schematic diagram of 2 E and 4 T1 energy level transitions doped with Cr^3+^ and Fe^3+^ in crystals. (**d**) Histogram of the percentage error of different algorithms predicting the results of the materials. Reproduced from Ref. [101] with permission from the Royal Society of Chemistry.

In terms of key modeling techniques, Ding et al. reported supervised models for Mn^4+^ hosts and, via recursive feature elimination over 32 structural descriptors, built a KNN regressor that accurately predicts peak emission wavelength from sparse literature data, as shown in Figure 10a,b [100]. For Cr^3+^/Fe^3+^ systems, Li et al. reported a regression workflow that learns the ^2E and ^4T_1_ level positions across oxide hosts with ≈1% error, enabling host–dopant tuning of NIR/red output, as shown in Figure 10c,d [101]. Together these studies show that carefully engineered crystal-field and lattice descriptors enable reliable forward prediction across Mn/Cr/Fe activators.

With respect to emerging architectures and algorithms, Ding et al. reported a model-comparison suite (KNN, SVR, RF, etc.), highlighting nonparametric and ensemble learners as robust baselines for small, heterogeneous datasets [100]. Complementing this, Wang et al. reported a random-forest pipeline trained on known Mn^4+^ fluorides; feature-importance analysis guided synthesis of Cs_2_NaAlF_6_:Mn^4+^ with ultra-narrow 628 nm emission and 99.7% color purity, validating ML-informed design in experiment [102].

Regarding data efficiency strategies, To mitigate data scarcity, Ding et al. reported screening of 278 ICSD-derived candidates with their KNN model, followed by selective synthesis of six top predictions—all confirmed experimentally, thus maximizing information per experiment [100]. Li et al. reported robustness to noisy, limited labels by favoring regularized/robust regressors that preserved sub-1% spectral-level errors [101]. Wang et al. reported an ML-triaged synthesis loop in which RF-ranked hosts were prioritized for fabrication, shortening the path from hypothesis to validated Mn^4+^ emitters [102]. Collectively, supervised feature engineering (Ding; Li), resilient learners, and selective validation (Wang) constitute a practical CEJ-style playbook for accelerating Mn^4+^/Cr^3+^/Fe^3+^ phosphor discovery, as shown in Figure 10a–d.

### 3.5. Perovskite Luminescent Materials

Perovskite emitters—including colloidal CsPbX_3_ (X = Cl/Br/I) nanocrystals and layered hybrids—offer high PLQY and tunable band gap, but are sensitive to composition and processing; ML is increasingly used to map composition–process–structure to emission color, yield, and stability in a data-efficient, designable manner.

In terms of key modeling techniques, Cakan et al. reported an interpretable workflow that links time-evolving PL/spectral descriptors of triple-halide films to durability and implements a regression–Bayesian predictor under light/heat stress [103]. Wu et al. introduced a data-driven band-gap resource to analyze halide segregation and trained predictors that generalize across compositions, enabling band-gap/emission trend forecasting from heterogeneous literature and computed data [104]. A physics-guided structured Gaussian-process surrogate with chemically informed mean functions was reported to improve band-gap targeting with calibrated uncertainty, directly informing emission-color design [105].

**Figure 11:**
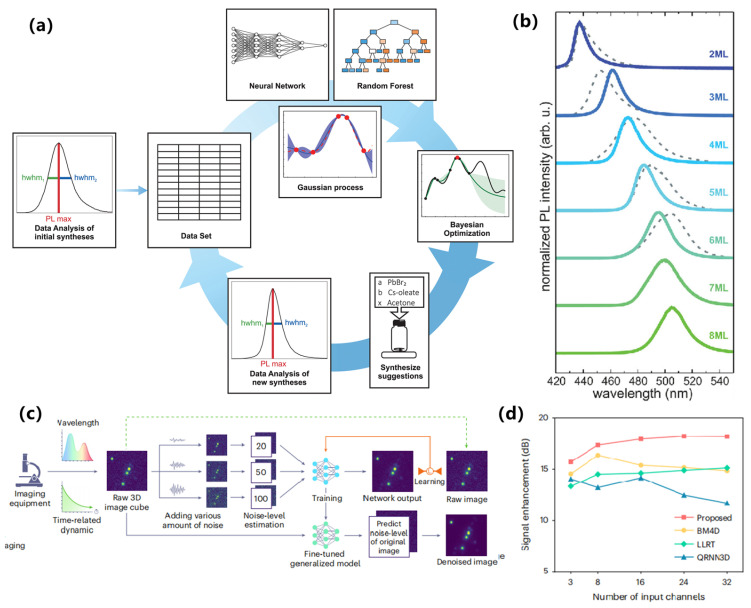
(**a**) Scheme of the optimization process: Initially existing data points (syntheses) were analyzed and used to predict a spectral figure of merit (FoM) based on the narrowness and symmetry of their PL spectra using Gaussian processes in combination with a random forest and a neural network. (**b**) Optimized PL spectra for all NPL thicknesses (colored lines) compared to initial, typical PL spectra (dashed gray). As the seven and eight ML NPLs were only obtained through optimization, there are no initial spectra. Reproduced from Ref. [106] with permission from the Wiley-VCH GmbH. (**c**) A convolutional residual network with a universal noise-level estimator. The noise level value corresponds to the s.d. of Gaussian noise. (**d**) The PSNR improvement after denoising against the number of channels (*n* of the input image (σ = 20). Reproduced from Ref. [108] with permission from Springer Nature.

With respect to emerging architectures and algorithms, Lampe et al. merged supervised models with Bayesian optimization to steer CsPbBr_3_ nanoplatelet syntheses from precursor-ratio inputs, tuning the PL maximum toward cyan–green emission, as shown in Figure 11a,b [106]. Gu et al. introduced a GNN-based synthesizability classifier (PU-learning + transfer) that achieves high out-of-sample true-positive rates, enabling high-throughput triage of feasible halide perovskites prior to optical screening [107]. At the platform scale, Omidvar et al. coupled ML screening with robotic synthesis and high-throughput characterization to accelerate exploration of solid-solution spaces in a closed loop from proposals to measured properties, as shown in Figure 11c,d [108].

**Figure 12:**
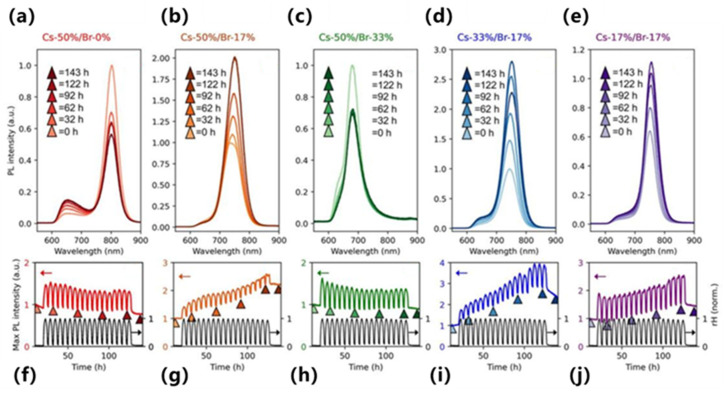
Effect of humidity cycling on photoluminescence. PL spectra for samples (**a**) Cs-50%/Br-0%, (**b**) Cs-50%/Br-17%, (**c**) Cs-50%/Br-33%, (**d**) Cs-33%/Br-17%, and (**e**) Cs-17%/Br-17% at five time points during the 118 h experiment (corresponding to 18 rH cycles). Each spectrum is acquired under identical environmental conditions (22 °C, rH < 5%) after 0, 32, 62, 92, 122, and 143 h. Maximum PL intensity for samples (**f**) Cs-50%/Br-0%, (**g**) Cs-50%/Br-17%, (**h**) Cs-50%/Br-33%, (**i**) Cs-33%/Br-17%, and (**j**) Cs-17%/Br-17% subjected to relative humidity (rH) cycling for 108 h (total experiment time is 144 h). The normalized rH profile is shown (black line) on each plot, see right y-axis. The left y-axis denotes the normalized PL. Each 6 h cycle ranges from <5 to 70% rH. The color-coded arrows correspond to the selected spectra shown in (**a**–**e**).

Regarding data efficiency strategies, A microfluidic auto-meta-learner (AMML) with coiled-flow reactors was used to synthesize cesium lead-halide nanocrystals at room temperature, leveraging meta-learning to reach target emission with few experiments [109]. Active meta-learning in J. Chem. Phys. combined uncertainty-aware learners with autonomous selection to prioritize informative candidates and reduced simulation/experimental demands for band-gap ranking (and implied emission colors) [110]. Physics-driven GP surrogates with crystal-chemistry-aware priors demonstrated data-efficient band-gap optimization with calibrated uncertainty; practical stability mapping under humidity cycling is shown in Figure 12 [111]. Beyond guiding the synthesis of emitters, ML models also excel at predicting fundamental structural properties that underpin optical performance. Alfares et al. employed Gaussian Process Regression (GPR) to achieve exceptional accuracy (R^2^ > 0.99) in predicting the lattice constants of ABX_3_ perovskite materials using basic elemental descriptors like ionic radii and electronegativity [112]. This capability allows for researchers to virtually screen and down-select promising perovskite compositions with desired structural parameters before embarking on resource-intensive experimental synthesis or DFT calculations, significantly accelerating the discovery pipeline for novel optoelectronic materials. Summary, Interpretable/physics-guided surrogates, GNN-aided feasibility screening, and Bayesian/active/meta-learning are converging into closed-loop workflows that predict and optimize PL color and yield with minimal measurements, enabling inverse-design strategies for perovskite emitters.

### 3.6. Others Organic/Small-Molecule Fluorescent Materials

Small-molecule fluorophores underpin sensing, bioimaging, and display applications, yet diverse backbones, conformational ensembles, and solvent/polarity effects complicate rational optimization of emission color, quantum yield, and Stokes shift. Machine learning (ML) provides data-driven mappings from molecular structure and environment to spectral outputs, enabling faster triage and design.

**Figure 13:**
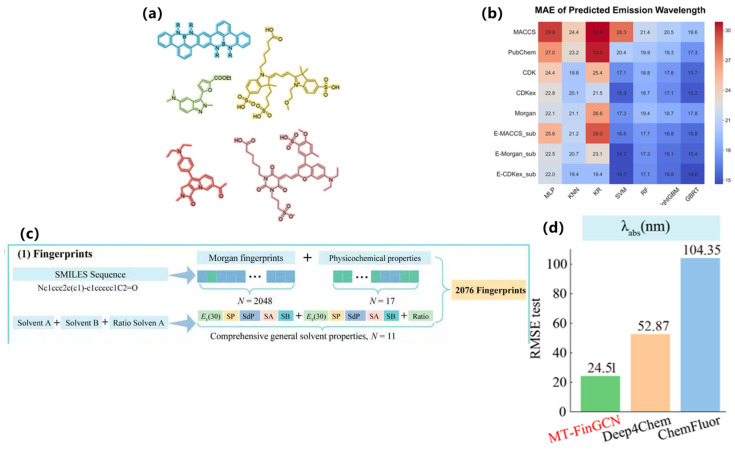
(**a**) Selective organic dyes in our database. (**b**) Testing results of emission wavelength of different combinations of ML models with different structure-based descriptors as inputs. Reproduced from Ref. [65] with permission from American Chemical Society; (**c**) flowchart of the NIRFluor’s fingerprints’ preparation. (**d**) Performance of MT-FinGCN and two existing SOTA models. Reproduced from Ref. [118] with permission from American Chemical Society.

In terms of key modeling techniques, Supervised and interpretable pipelines now predict fluorescence metrics directly from structure/solvent descriptors. Souza et al. trained models on the Deep4Chem corpus (20,236 molecule–solvent combinations) to jointly predict emission wavelengths and QYs, showing robust generalization across chemotypes and media [113]. Mahato et al. developed hybrid ensemble regressors over 3066 organic dyes to estimate absorption/emission wavelengths and QY in a single workflow, illustrating how model stacking improves accuracy for multi-property prediction [114]. For mechanistic transparency, Chebotaev et al. used QSPR/ML on BODIPY photosensitizers to predict the fluorescence-to-singlet-oxygen generation ratio and ranked key descriptors (e.g., electronic and topological terms) that govern competition between radiative and photochemical channels [115]. As shown in Figure 13a,b, model–descriptor combinations for organic dyes yield accurate emission-wavelength predictions across the curated dye database [65].

With respect to emerging architectures and algorithms, new model classes support inverse design and richer structure–property learning. Han et al. introduced a generative deep-learning framework to design small organic fluorophores at target optical properties, demonstrating constrained generation guided by learned structure–optics rules [110]. Jung et al. combined deep residual CNNs with solvent encoding to predict peak optical absorption from SMILES, offering an architecture readily extensible to fluorescence endpoints and solvent effects [116]. At the probe level, Xiang et al. applied ML-assisted design to xanthene-type Si-rhodamine systems, quantitatively linking substituent patterns to pH responsiveness and in situ imaging SNR, and using the model to guide synthesis of a high-performance probe [117]. As shown in Figure 13c,d, Wang et al. demonstrated that the NiRFluor multitask FinGCN platform markedly improves multi-endpoint prediction for NIR small-molecule fluorophores [118].

Regarding data efficiency strategies, to counter sparse, heterogeneous labels, groups exploit literature-mined corpora, curated benchmark sets, and physics-guided targets. Zhu et al. built a modular AI framework that taps text-mined optical data (e.g., the ChemFluor database, >4300 solvated fluorophores) to pretrain predictors before task-specific fine-tuning, improving data efficiency for fluorophore discovery [119]. Shao et al. released SMFluo1 (1181 solvated fluorophores) and trained deep models to predict λ_max, establishing solvent-aware baselines that transfer to related fluorescence tasks [120]. Complementing purely statistical learners, Ravasco et al. used physics-grounded multilinear free-energy relationships (mLFER) as a compact surrogate to discover a new BASHY dye with targeted emission—showcasing how mechanistic priors reduce data needs while preserving designability [121]. Altogether, for general organic fluorophores, enhancement strategies (multi-property supervised learning with interpretable features) deliver reliable, explainable predictions; emerging architectures (generative models, solvent-aware deep networks) enable inverse design across chemotypes; and data-efficiency tactics (literature-mined corpora, curated solvent datasets, physics-guided surrogates) mitigate label scarcity. Together, these advances convert empirical dye tuning into principled, ML-assisted design workflows with clear routes from molecular blueprint to emission performance.

The aforementioned data-driven approaches are widely applicable to various fluorescent materials, among which benzimidazole-based fluorescent probes exhibit strong binding affinity for heavy metal ions and thus can be employed for water quality testing and environmental monitoring [122]. Machine learning (ML) can significantly accelerate the optimization of such probes; for instance, by predicting the positions of substituents, it enables the adjustment of emission wavelengths and quantum yields to generate distinct signals, thereby improving the reliability of analyte detection [123,124]. Integrating ML into the design of these systems is highly beneficial for developing sensors with high sensitivity and high selectivity. This approach holds great promise for directly contributing to societal well-being through advanced environmental monitoring.

### 3.7. Iridium III/Platinum II Complex Phosphors

Iridium(III) and platinum(II) phosphors remain workhorse emitters for OLEDs because strong spin–orbit coupling enables efficient triplet harvesting and color tuning across the visible–NIR, yet optimizing radiative rates, lifetimes, and spectral shape still demands costly synthesis–DFT cycles; recent ML methods increasingly bridge this gap from structure to photophysics and device-relevant figures of merit [125,126].

**Figure 14:**
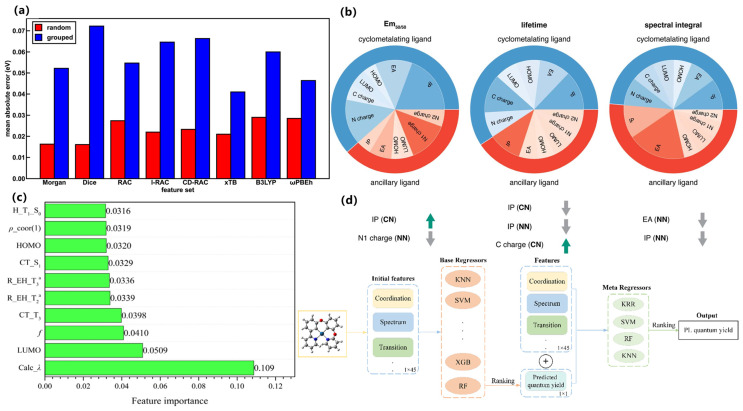
(**a**) The test set performance of ANNs trained on different feature sets in predicting Em50/50 (MAE, in units of eV) for both random (red bars) and grouped splits (blue bars). Here, l-RAC refers to ligand-only RACs. (**b**) For each of the three target properties, the corresponding column indicates: (top) random forest feature importances of the xTB CN and NN features and (bottom) the correlation of the most important xTB features to the target property, where a green arrow indicates positive correlation and a gray arrow indicates negative correlation. (**c**) Ten most important features for emission wavelength extracted from LightGBM-based machine learning model. (**d**) Stacking architecture for photoluminescence quantum yield prediction. Reproduced from Ref. [127] with permission from Wiley Periodicals LLC.

In terms of key modeling techniques, Low-cost, interpretable prediction of excited-state observables has become feasible for large Ir(III) datasets. Terrones et al. trained models on electronic-structure features from tight-binding calculations to predict mean phosphorescence energy, excited-state lifetime, and emission spectral integral for 1380 Ir complexes with accuracy competitive with or exceeding TDDFT, while feature analyses linked ligand ionization potentials to color and lifetime trends [127]. Pal et al. used deep learning to predict full emission spectra of heteroleptic Ir(III) complexes, capturing host/ligand effects on spectral envelopes and enabling spectrum-aware screening rather than single-point wavelength fits [128]. Complementing these, Karuth et al. combined QSAR, computation, and experiment to relate structural variation to red-to-near-IR emission in Ir(III) complexes, establishing data-driven rules that map substituent changes to spectral shifts [129]. It can be observed from Figure 14a,b that Zhao et al. applied neural networks and ensemble models to Ir(III)/Pt(II) phosphors, revealing ligand ionization energy as a dominant factor in emission tuning [127].

With respect to emerging architectures and algorithms, Methodological breadth now includes stacked/ensemble learners and HTVS pipelines tailored to organometallic emitters. Peng et al. reported stacking and multitask models that jointly predict photophysical properties (e.g., emission and absorption wavelengths) of Ir(III) complexes, improving generalization over single learners and consolidating property prediction into one workflow [130]. For Pt(II) systems, Wang et al. built an ensemble ML framework to predict photophysical properties from structural descriptors, demonstrating robust accuracy across diverse square-planar complexes and providing a transferable screening surrogate [131]. At larger scale, a Materials Futures study introduced a general protocol that couples generation of >3600 Pt(II) complexes with HTVS and ML surrogates to predict emission energy, S1–T1 gaps, and non-radiative rate constants, accelerating down-selection toward high-PLQY candidates [132]. As shown in Figure 14c,d, a LightGBM model extracts the top features for emission wavelength, while a stacked architecture improves PLQY prediction [133].

Regarding data efficiency strategies, Physics-guided targets, representation learning, and semi-automated synthesis reduce labeling burdens while preserving fidelity. Hatanaka et al. replaced hard-to-measure LQY with a mechanistic proxy—the energy gap between six- and five-coordinate triplet structures—then combined DFT with ML to rationalize non-radiative pathways in cyclometalated Ir(III), yielding interpretable, data-efficient predictors of brightness [134]. Cheng et al. performed representation-learning-aided HTVS over millions of Ir(III) complexes assembled from 278 known ligands, screening unsynthesized emitters prior to costly computations or experiments and thus amortizing discovery over a vast virtual library [31]. In parallel, a semi-automated, high-throughput platform produced a 90-member Ir(III) library with standardized measurements, creating high-quality corpora that can seed active-/transfer-learning loops for future ML models [135]. Taken together, Across Ir(III)/Pt(II) phosphors, enhancement strategies (task-specific regressors, spectrum-aware deep models) deliver accurate, interpretable predictions; emerging algorithms (stacked/ensemble surrogates and HTVS protocols) scale screening beyond heuristic design; and data-efficiency tactics (mechanistic proxies, representation learning, semi-automated datasets) make discovery tractable with limited labels—together enabling faster, more reliable translation from ligand sets to device-grade emitters.

### 3.8. ns^2^ Metal Halides (e.g., Bi^3+^/Sb^3+^)

Rare-earth–free ns^2^ metal halides featuring Bi^3+^ or Sb^3+^ activators offer broadband, large-Stokes-shift emission from self-trapped excitons with promising stability and low toxicity; however, their photophysics are exquisitely sensitive to local coordination, dimensionality, and composition, making them ideal targets for machine-learning (ML) models that can link structure, processing, and emission behavior [136].

In terms of key modeling techniques, Supervised and interpretable pipelines have begun to quantify how local structure governs emission efficiency and dynamics in Bi^3+^/Sb^3+^ systems. Molokeev et al. used principal-component analysis and random-forest models across a curated library of zero-dimensional ns^2^ halides to show that concentration quenching and cation–cation distances dominate PLQY trends, with secondary roles from site symmetry and polyhedral distortion; the model rationalized host choices and guided discovery of brighter emitters [137]. Building on lifetime–efficiency correlations, Yang and Wang trained multi-model regressors to predict fluorescence lifetimes of zero-dimensional antimony halides, attaining low error using purely structural features and enabling rapid screening of synthesis targets [138]. For hybrid Bi/Sb halides where templating cations steer framework motifs, Blahusch et al. developed an interpretable ML classifier/regressor to learn templating effects and forecast the formation of specific inorganic substructures from organic-cation descriptors—thereby connecting design knobs to emergent emission-relevant connectivity [139].

With respect to emerging architectures and algorithms, new model classes and search strategies extend beyond hand-crafted descriptors to navigate broader composition–structure spaces. A learning-templatability workflow for hybrid antimony/bismuth halides combined feature-engineered text/graph inputs with ML to predict structure types before synthesis, shortening iteration cycles and enabling a priori selection of hosts likely to stabilize emissive zero-/low-dimensional networks [139]. In parallel, Bayesian/active optimization paradigms developed for halide perovskites are being adapted to ns^2^ halides to plan experiments efficiently—e.g., closed-loop optimization of processing or composition using PL-based figures of merit—offering data-efficient routes to tune emission color and stability [103]. Beyond scalar targets, data-driven microstructural optimization in Bi-containing halides illustrates how CNNs and BO can translate image-level observations into actionable synthesis guidance, a capability directly transferable to morphology-sensitive Bi^3+^/Sb^3+^ phosphors [140].

**Figure 15:**
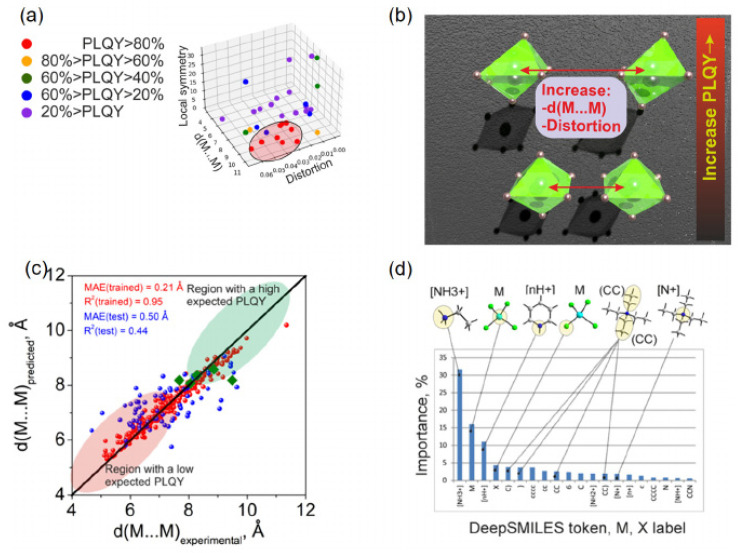
(**a**) Samples with high PLQY values (red circles in highlighted area) are segregated from others in the 3D space spanned on three most important parameters. (**b**) Importance of four feature parameters on PLQY values in the RF model. The d(M…M) distance has the major influence. Reproduced from Ref. [137] with permission from American Chemical Society. (**c**) Comparison between predicted and experimentally observed shortest d(M…M) distances in OIMH compounds. (**d**) Importance of first 20 feature parameters on d(M…M) obtained by Random Forest method. Reproduced from Ref. [142] with permission from American Chemical Society.

Regarding data efficiency strategies, given sparse, heterogeneous labels, groups are leveraging weak supervision, proxy targets, and physics-guided features. The Chem. Mater. dataset from Molokeev et al. provides open PLQY/structure records for ns^2^ halides that have been reused for feature screening and re-training, reducing the need for de novo measurements [137].For lifetime/efficiency prediction where high-fidelity kinetics are costly, structure-only models (Yang & Wang) act as surrogates to triage candidates prior to spectroscopy, while error-aware ensembles curb overfitting on small sets [138]. Finally, physics-guided descriptors tailored to ns^2^ activators—such as Duffy-type optical electronegativity, crystal-field/centroid-shift proxies, and dimensionality metrics—serve as informative priors that improve extrapolation and interpretability when labels are scarce [141]. As shown in Figure 15a,b, random-forest analysis links short metal–metal distances and related structural distortions to higher PLQY in ns^2^ metal halides [137]. As shown in Figure 15c,d, Molokeev et al. used a Random Forest/Decision Tree framework to predict the shortest M…M distance in OIMHs with a test MAE ≈ 0.5 Å and to rank [NH^3+^], [nH^+^], and aliphatic-branch tokens (‘C’) as key features, supporting an ≈ 8.0 Å threshold for high PLQY and enabling efficient screening of bright scintillators [142]. Overall, Across Bi^3+^/Sb^3+^ halides, enhancement strategies (interpretable supervised models tied to local geometry) provide actionable design rules; emerging algorithms (structure-type predictors, BO-driven experiment planning, microstructure-aware surrogates) expand exploration; and data-efficiency tactics (open datasets, proxy targets, physics-guided features) make discovery tractable under limited labels—together shifting ns^2^ halide development from empirical screening toward predictive, mechanism-aware design [137].

### 3.9. Group-III Nitride LED Materials (e.g., GaN, AlGaN)

Group-III nitrides underpin today’s visible (In/GaN) and deep-ultraviolet (AlGaN) LEDs, yet performance hinges on tightly coupled choices in epitaxy, quantum-well design, and defect control; machine learning (ML) is increasingly used to connect growth/process parameters and device structures to emission color, efficiency, and stability, enabling faster, more reliable optimization across this multi-variable space [143,144].

**Figure 16:**
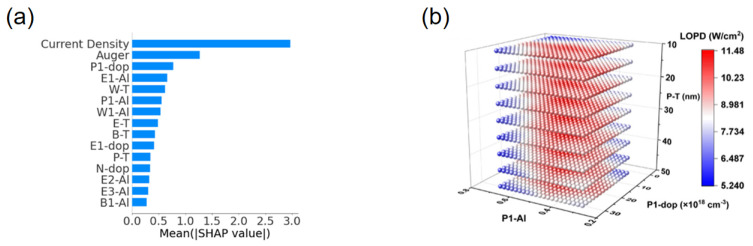
(**a**) The global importance of top 15 features based on the average SHAP value Magnitude. (**b**) Predicted LOPD with changing of different features:4D-scatter plot of LOPD with respect to P1-Al, P1-dop, and P-T.

In terms of key modeling techniques, Supervised, interpretable models now predict device-level metrics directly from structural/process descriptors. Jiang et al. used ML to design GaN-based LED architectures, accurately predicting performance, highlighting critical structural features, and screening >20,000 candidates within seconds—demonstrating direct, model-driven guidance for structure selection during manufacturing [145]. For AlGaN deep-UV emitters, Lin et al. trained four ML models on a curated multi-year dataset and showed a CNN yields the most accurate luminous-power predictions (R^2^ ≈ 0.98), while revealing which layer parameters most influence DUV LED output [146]. Complementing emitter design, Gallagher et al. built wafer-screening regressors for vertical GaN devices from non-destructive profilometry/optical data, an approach transferrable to LED lines for rapid correlation of material quality with device performance [147]. It can be seen from Figure 16a,b that SHAP-guided CNN modeling ranks key structural/process parameters and boosts predicted deep-UV nitride LED output [146].

With respect to emerging architectures and algorithms, Physics-informed and structure-aware learners are expanding the design/search toolkit. Kobayashi et al. introduced a physics-informed Bayesian optimization (PIBO) for compound-semiconductor MOCVD that embeds Vegard’s law and gas-flow/composition monotonicity; PIBO successfully synthesized target-bandgap films outside the training domain—an approach directly applicable to GaN/AlGaN growth windows where extrapolation is essential [59]. At the active-region level, Pant, Armitage, and Kioupakis trained ML surrogates on multi-scale quantum simulations and uncovered counter-intuitive benefits of polarization fields for red InGaN quantum wells (thinner wells via QCSE with higher overlap), yielding machine-learned design rules for long-wavelength nitrides [148]. On the metrology side, GaN micro-LED arrays analyzed by hyperspectral imaging coupled with ML enable automatic defect detection and yield triage at the pixel level—essential for scaling µLED manufacturing [149].

Regarding data efficiency strategies, Given the high cost of epitaxy/characterization, groups leverage label-efficient schemes. Physics-guided priors (e.g., Vegard-based targets and monotone gas-flow constraints in PIBO) reduce the number of growth trials while improving extrapolation to untested conditions [59]. Imaging-rich, weakly supervised pipelines—such as AOTF-hyperspectral analysis of GaN µLED arrays—extract informative labels from routine inline scans, minimizing destructive testing [149]. Finally, non-destructive feature sets (profilometry/optical) paired with supervised models enable fast wafer-level ranking before device fabrication, conserving resources while preserving predictive power for downstream LED metrics [147].Collectively, Across GaN/AlGaN LEDs, enhancement strategies (supervised predictors with interpretable features) deliver reliable performance estimates; emerging tools (physics-informed BO, polarization-aware surrogates, hyperspectral-ML inspection) unlock design spaces and scale to manufacturing; and data-efficiency tactics (priors, weak supervision, non-destructive inputs) make exploration tractable. Together these advances are moving nitride emitters from empirical tuning toward principled, ML-guided inverse design and process control [59,145,146].

### 3.10. Photovoltaic Materials Electrical-Performance Analysis (e.g., Si, GaAs)

Silicon and III–V (e.g., GaAs) photovoltaics dominate terrestrial and high-efficiency space power, yet extracting device parameters, diagnosing defects, and linking growth/processing to electrical figures of merit still require time-consuming measurements and expert analysis; machine learning (ML) now provides fast, accurate surrogates that turn raw I–V/IQE/EL data into actionable electrical performance insights.

**Figure 17:**
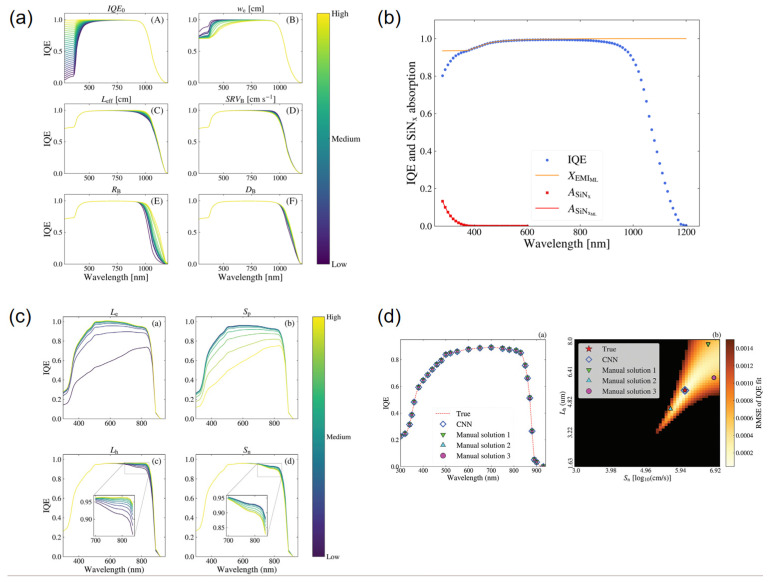
(**a**) Representation of the effect of individual parameters on the simulated internal quantum efficiency (IQE) data, (A) IQE0, (B) we, (C) Leff, (D) SRVB, (E) RB, and (F) DB. (**b**) Simulated/measured internal quantum efficiency (IQE) with ASiNx (blue dots) and the IQE curve generated considering only IQE0 and we, XEMIML (orange line). Reproduced from Ref. [153] with permission from John Wiley & Sons Ltd. (**c**) The effect of different parameters on the IQE. (**d**) A representative example of multiple solutions providing a similar fit: the true IQE, CNN prediction, and manual fits and the RMSE heatmap. The red star identifies the true values while the blue open diamond denotes the CNN model’s prediction. Reproduced from Ref. [151] with permission from Wiley-VCH GmbH.

In terms of key modeling techniques, Tang et al. inferred microscopic parameters of silicon heterojunctions directly from measured I–V curves with a trained deep model, recovering junction/interface descriptors that traditionally demand iterative fitting and thereby enabling rapid, non-destructive electrical characterization [150]. For GaAs, Abdullah-Vetter et al. mapped internal-quantum-efficiency (IQE) spectra to multiple key electrical parameters using a noise-robust deep network, achieving high accuracy over the full parameter range and resilience to measurement noise [151]. At the module level, Liu et al. trained a convolutional model on electroluminescence (EL) images to detect cell-level defects efficiently; contrast-enhanced inputs and a lightweight CNN delivered precise defect flags that correlate with power loss, supporting fast pass/fail decisions in production [152]. As shown in Figure 17a,b, random forests and CNNs reliably extract internal-quantum-efficiency parameters from spectra, outperforming manual fitting under noise [153]. It can be observed from Figure 17c,d that sensitivity analyses and error-landscape maps reveal parameter influence and degenerate solutions yielding similar IQE curves [151].

With respect to emerging architectures and algorithms, Physics-informed search and specialized deep networks are expanding capability beyond conventional regression. Kobayashi et al. embedded Vegard-type priors and monotonic gas-flow/composition constraints into Bayesian optimization for III–V MOCVD, successfully extrapolating to target bandgaps outside the training domain—an approach that directly translates to GaAs device-grade growth for electrical-performance targets [59]. He et al. deployed a two-stream DNN that fuses I–V curve “images” with engineered features to diagnose shading type and severity, improving array-level fault classification and enabling automated recovery strategies [154]. Complementarily, Abdelsattar et al. used deep residual networks (ResNet-34/50/152) on EL imagery to detect micro-/macro-cracks with F1 up to ~0.89, providing a robust architecture for electrical-reliability triage tied to crack-induced series-resistance penalties [155].

Regarding data efficiency strategies, to curb labeling cost and improve robustness, groups combine weak supervision, augmentation, and fieldable data acquisition. Daylight-EL methodologies reviewed by del Prado Santamaría et al. extend EL imaging to outdoor conditions using InGaAs cameras, filtering, and current modulation, vastly expanding real-world datasets for defect-to-performance learning without lab setups [156]. Pan et al. reduced false positives in EL-defect detection with an adaptive complementary-fusion (ACF) module that integrates spatial and channel cues, improving accuracy under limited annotations and heterogeneous image quality [157]. For GaAs IQE analysis, Abdullah-Vetter et al. bootstrapped supervised models with simulation-derived spectra and then injected realistic noise to harden the predictor, demonstrating a practical weak-label route toward parameter extraction when experimental labels are scarce [153]. Collectively, Across Si and GaAs photovoltaics, enhancement models now infer device parameters from I–V/IQE and flag EL-visible defects with production-grade speed; emerging algorithms—physics-informed Bayesian optimization and task-specific deep nets—bridge growth/process decisions to electrical performance; and data-efficiency tactics (daylight EL, simulation-augmented labels, robust fusion blocks) scale learning to realistic conditions. Together these advances transform electrical-performance analysis from iterative expert fitting into rapid, ML-assisted diagnostics and process control.

### 3.11. Semiconductor Quantum Dot

Semiconductor quantum dots (QDs) span II–VI (e.g., CdSe/CdS), IV–VI (e.g., PbS/PbSe), III–V (e.g., InP), and emerging MXene/carbon-dot families, offering size- and composition-tunable emission across the visible–NIR; machine learning (ML) now links synthesis, structure, and spectra to accelerate color/QY targeting and device translation.

**Figure 18:**
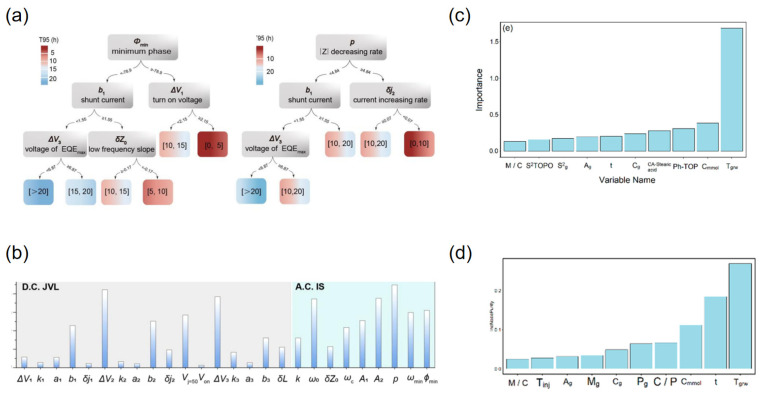
(**a**) Classification decision tree with the structures of five-level decision tree and selected five features: *ϕ*min, *b*1, Δ*V*1, Δ*V*3, and *δZ*0; and structure of the three-level decision tree and selected four features: *p*, *b*1, *δj*2, and Δ*V*3. (**b**) Results of Pearson correlation analysis for all features with QLED lifetime. Reproduced from Ref. [161] with permission from American Chemical Society. (**c**) Importance of the variables in the random forest regression model. (**d**) Importance of the variables in the gradient-boosting machine regression model. Reproduced from Ref. [165] with permission from American Chemical Society.

In terms of key modeling techniques, Nguyen et al. learned direct mappings from InP-QD synthesis descriptors to absorption, emission, and diameter, enabling supervised property prediction for heavy-metal-free III–V dots [158]. Malashin et al. modeled temperature-dependent PL of II–VI CdS QDs with LSTM sequence learners, capturing time/temperature-linked fluctuations that frustrate conventional fits [159]. For application-level screening, Corcione et al. combined spectral feature extraction with an autoencoding CNN to classify single-photon suitability from emission traces of semiconductor QDs, illustrating interpretable, data-driven triage [160]. As shown in Figure 18a,b, decision-tree structures and Pearson correlations expose descriptors that dominantly affect QD-LED lifetime [161].

**Figure 19:**
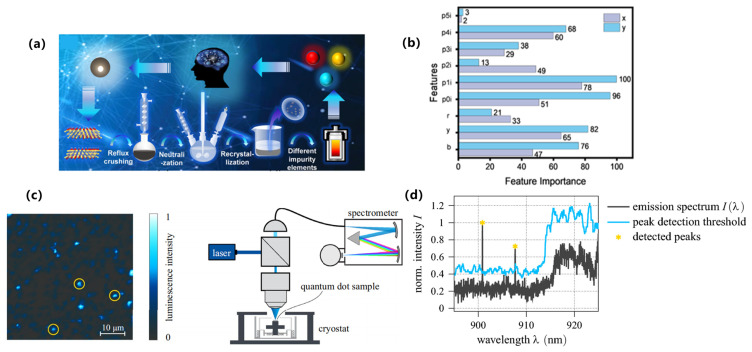
(**a**) Schematic of machine learning guided full-color MQDs used to build WLEDs; (**b**) feature importance of nine features generated from the XGB model. Reproduced from Ref. [166] with permission from Royal Society of Chemistry. (**c**) Schematic measurement setup: The QD sample is placed inside a Helium cryostat and excited by an above band laser guided through a beamsplitter. The luminescence signal is collected and sent to a spectrometer. Three exemplary QDs are marked in yellow. (**d**) Working principle of the OS-CFAR peak detection algorithm 51. Data points above the adaptive threshold are identified as spectral peaks. Reproduced from Ref. [160] with permission from Springer Nature.

**Figure 20:**
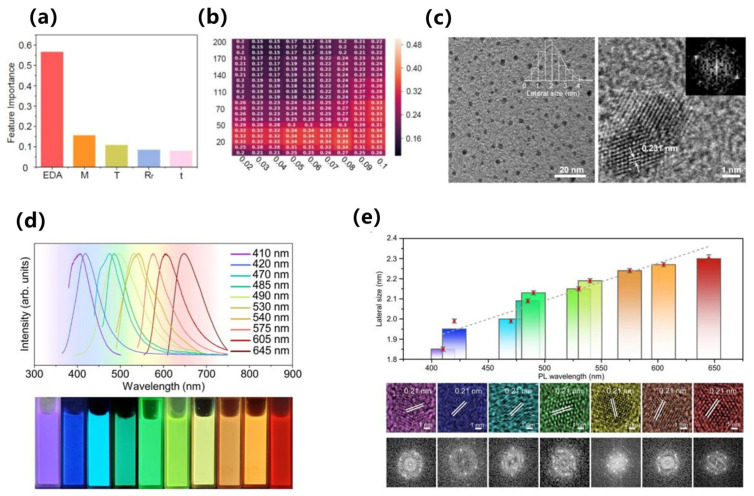
(**a**) Feature importance retrieved from XGBoost-R that learns from the full dataset. The most important features are EDA and M. (**b**) Predictions from the trained model, which is represented by the matrix formed by the two most important features. (**c**) Photographs of CQDs under 365 nm-UV light irradiation and dependence of the HOMO and LUMO energy levels of CQDs. Reproduced from Ref. [166] with permission from American Chemical Society. (**d**) Morphological characterizations and relationship analysis between synthesis parameters and optical properties of full-color fluorescent CQDs. Reproduced from Ref. [167] with permission from Springer Nature. (**e**) The lateral size and color of full-color fluorescent CODs (inset: dependence of thePl wavelength and the lateral size of full-color fluorescent CODs). Data correspondto mean± standard deviation, n = 3. High-resolution TEM images and the fastFourier transform patterns of p-, b-, c-,g-, y-, o- and r-CODs, respectively.

With respect to emerging architectures and algorithms, Allara et al. fused wide-angle X-ray total scattering with a deep classifier to size-classify PbS QDs (IV–VI) without calibration curves, unifying structural/microstructural cues for robust sizing [162]. Park et al. closed the loop for II–VI CdSe in an oscillatory-flow microreactor, using Bayesian/ML optimization with in situ optics to converge on target extinction/emission in few iterations [163]. For III–V nanowire QDs, Zieliński et al. coupled atomistic tight-binding with neural networks and transfer learning to recover ground-state energies of double InAs/InP QDs with ~1 meV RMSE, pointing to spectrum-aware inverse design [164]. As shown in Figure 18c,d, random-forest and gradient-boosting regressors provide complementary variable-importance profiles across device/material features [165]. As shown in Figure 19a,b, ML-guided material design achieves full-color MQDs for WLEDs, with XGBoost feature importance guiding emission control [166]. It can be seen from Figure 19c,d that an instrumented measurement setup and OS-CFAR peak detection enable robust, high-throughput spectral characterization [160]. As shown in Figure 20a–c, XGBoost-R highlights key synthesis descriptors (e.g., EDA), visualizes accurate predictions over top-feature matrices, and illustrates full-spectrum CQD emission (UV-excited photographs and HOMO/LUMO relations) [167]. It can be observed from Figure 20d,e that morphology characterizations and feature–property relations explain how synthesis parameters steer optical properties across full-color CQDs [168].

Regarding data efficiency strategies, Guo et al. achieved full-color, high-QY carbon dots via multi-objective ML-guided hydrothermal synthesis, using limited experiments to optimize color/QY/stability trade-offs [168]. El-Azazy et al. reviewed literature-mined corpora and weak-label strategies for CQDs—frameworks that pretrain models on heterogeneous optical data before task-specific fine-tuning [169]. For MXene QDs, Lin et al. implemented ratiometric sensing with smartphone readout; while primarily analytical, such platforms create scalable, image-based labels that can seed future ML predictors for emission/intensity normalization [170]. Summary, Across II–VI/IV–VI/III–V and emerging MXene/carbon-dot families, enhancement models (supervised, interpretable, sequence-aware) deliver reliable PL/size predictions; architectures and algorithms (deep classifiers with structural probes, closed-loop BO, ML-accelerated atomistics) enable sizing, color targeting, and energy-level tuning; and data-efficiency (multi-objective optimization, literature-mined pretraining, smartphone-scale labeling) makes exploration tractable—turning QD discovery from trial-and-error into ML-guided, figure-of-merit-driven design Figure 18, Figure 19 and Figure 20.

### 3.12. Machine Learning of Spectrum Identification/Spectrum–Performance Mapping

Optical spectra (PL/EL/absorption/Raman and hyperspectral cubes) encode rich fingerprints of composition, defects, and excited-state dynamics; machine learning (ML) now turns these signals into fast identification and quantitative links to device-level performance across emitters and optoelectronic stacks.

In terms of key modeling techniques, Supervised and interpretable pipelines have matured for spectrum identification and basic property inference. Qi et al. used a CNN paired with diffusion-model denoising to classify 2D materials from Raman spectra with ~98–99% accuracy across substrates, demonstrating robustness to spectral variability and practical noise (spectrum → material ID) [171]. Wang et al. proposed a deep Raman identification framework that borrows convolutional feature extractors (RST), improving qualitative analysis over classical chemometrics (spectrum → phase/class) [172]. Beyond hard labels, Lu et al. aggregated weak, noisy Raman signals with a deep learner to identify cellular phenotypes, an approach transferable to materials defect states where peaks shift/broaden under processing (spectrum → state) [173].

With respect to emerging architectures and algorithms, new model classes target harder spectrum tasks—artifact removal, temporal dynamics, and end-to-end performance mapping. The RADAR toolkit introduced lightweight networks that simultaneously denoise and correct Raman spectra, stabilizing downstream classifiers/regressors (spectral preprocessing → reliable ID) [173]. Laufer et al. coupled deep learning with time-resolved PL/GI-WAXS during large-area perovskite film formation to extract kinetic features predictive of film quality—turning spectral evolution into process control signals (spectral dynamics → quality index) [174]. At the performance end, Shi et al. integrated functional intensity-modulation two-photon spectroscopy with ML regression to map spectral-response patterns to excited-state parameters, offering a route from spectra to mechanistic figures of merit (spectrum → rate constants) [175].

**Figure 21:**
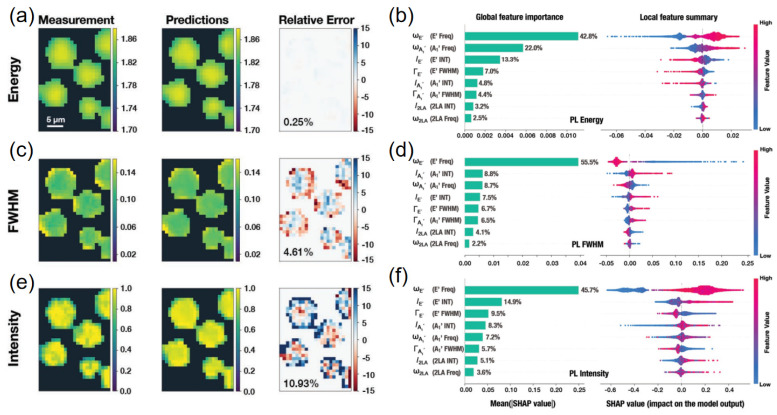
(**a**–**f**) the PL mapping predictions and Correlation analysis for Raman and PL by XGBoost with SHAP values of energy (**a**,**b**), FWHM (**c**,**d**), and intensity (**e**,**f**) for CVD-grown MoS_2_ with random shape. Reproduced from Ref. [180] with permission from Wiley-VCH GmbH.

Regarding data efficiency strategies, because curated spectral labels are scarce and heterogeneous, groups combine transfer/weak supervision with fast spectral acquisition. Shin et al. showed transfer learning halves data needs when predicting OLED optical properties and helps bridge simulation–experiment gaps with only a few dozen measured spectra (pretrain → fine-tune on real EL/PL) [176]. Hardware advances expand low-cost labels: LED- or DMD-based hyperspectral imagers deliver real-time cubes that feed weakly supervised learners for inline classification and mapping (rapid HIS → more labels) [177,178].Comprehensive reviews in Raman/perovskite optoelectronics summarize augmentation and semi-supervised tactics—text-mined spectra, physics-guided priors, and domain-adaptive training—to stabilize models across instruments and labs (literature/physics priors → robust generalization) [179]. Altogether, in spectrum-centric workflows, enhancement methods (robust CNNs with denoising and interpretable features) deliver high-accuracy identification; emerging algorithms (artifact-aware nets, temporal spectral learners, spectroscopy-to-mechanism regressors) push toward direct spectrum → performance links; and data-efficiency (transfer/weak supervision plus fast HSI acquisition and physics priors) makes models portable and economical. Together these advances convert spectra from qualitative fingerprints into quantitative, deployable predictors for materials selection, process control, and device benchmarking. As shown in Figure 21a–f, XGBoost with SHAP maps Raman features to PL energy, width, and intensity in monolayer MoS_2_, offering interpretable, data-efficient spectral–optical correlations [180].

Furthermore, the analytical sensitivity of fluorescence-based sensing, quantified by the limit of detection (LoD), can be substantially enhanced through ML approaches. By analyzing complex, multidimensional spectral data—such as subtle shifts in peak position, changes in full width at half maximum, or relative intensity variations across multiple peaks—ML models can identify faint but consistent signatures of analytes that are imperceptible to conventional analysis [181]. This capability allows for ML to effectively increase the signal-to-noise ratio, enabling the reliable detection of ultralow analyte concentrations. Consequently, ML-guided design of fluorescent probes and advanced spectral analysis paves the way for developing next-generation sensors with dramatically improved LoD for applications in environmental monitoring and clinical diagnostics.

### 3.13. Cross-System Comparative Analysis and Insights

A comparative analysis across the diverse fluorescent material systems reveals how the inherent nature of the material dictates the focus, methodology, and challenges of applying ML.

For organic molecular systems (e.g., AIEgens, TADF emitters, small-molecule fluorophores), the structure-property relationship is paramount. The primary data source is often computational molecular descriptors or graph representations of the molecular structure itself. Consequently, supervised learning models trained on these features excel at predicting intrinsic photophysical properties like emission energy and quantum yield. GNNs are particularly powerful here as they natively operate on molecular graphs, automatically learning relevant sub-structural features. The main challenge is the vastness of the chemical space, making exhaustive exploration impractical. ML’s role is to navigate this space efficiently, moving from empirical trial-and-error to rational molecular design.

In contrast, for inorganic and hybrid systems—such as quantum dots, perovskites, and phosphors—their optical properties are less susceptible to the influence of individual molecules, and are more strongly governed by synthesis conditions, compositions, dopant environments, and microstructures. Here, machine learning (ML) models typically correlate experimental factors (e.g., synthesis parameters, compositions, and ratios) with the final optical outputs. Bayesian Optimization (BO) demonstrates excellent performance in these contexts: it guides experimental synthesis to achieve optimal conditions with the minimum number of trials. The challenges in this work often lie in the high cost required to ensure the consistency of generated datasets, as well as the difficulty in capturing complex and elusive multivariate interactions.

Furthermore, there exists a universal challenge in this field, namely data scarcity and heterogeneity, though its manifestations differ across systems. In organic chemistry, the core issue is the vast combinatorial space of molecules, which leads to exorbitant trial-and-error costs. In inorganic chemistry, by contrast, a major challenge stems from the large scale and complexity of experiments—these require the synthesis and analysis of multiple substances under strictly controlled conditions. Consequently, tailored ML strategies are necessary: the former (for organic systems) leverages molecular representations, while the latter (for inorganic systems) focuses on process optimization rooted in solid-state chemistry.

Additionally, enhancing model interpretability is crucial for advancing scientific discovery and fostering researchers’ trust. Looking ahead, the most promising direction lies in the tight integration of machine learning with automated experiments. This integration establishes a closed-loop system, enabling rapid validation of predictions and iterative learning from results. Future efforts should also prioritize the development of more hybrid models—models that integrate the physical principles specific to each material class, moving beyond purely data-driven approaches to achieve more reliable and versatile design frameworks.

## 4. Challenges and Future Directions

Despite substantial progress in applying machine learning to luminescent materials, several fundamental challenges persist that limit its broader impact. These recurring issues—spanning data, models, and integration—must be addressed to fully realize the potential of ML-driven discovery in diverse emitter classes.

Data scarcity and heterogeneity: A cross-cutting obstacle is the paucity of high-quality, standardized data across luminescent material classes. Whether for novel AIE fluorophores, TADF emitters, or rare-earth phosphors, available datasets are often small and fragmented, with measurements conducted under inconsistent conditions. This scarcity and heterogeneity hamper model training and transferability, underscoring the need for community-curated databases and unified metadata schemas to aggregate experimental results. Consistent reporting standards and data-sharing practices would improve interoperability, while strategies like data augmentation, transfer learning, and multi-modal data integration can help mitigate limited data regimes.

Generalization and uncertainty: ML models for luminescence frequently exhibit narrow domain generalization, performing well on known chemistries but faltering on new compositions or structures. This issue spans both organic and inorganic luminophores—a predictor trained on a specific family (e.g., TADF molecules or perovskite nanocrystals) may struggle to extrapolate beyond that domain. Ensuring that models are robust and aware of their uncertainty is critical for practical deployment: without calibrated uncertainty estimates, experimentalists cannot gauge whether a predicted high-efficiency QD or OLED dye is a reliable candidate. Future approaches should emphasize rigorous uncertainty quantification (e.g., Bayesian or ensemble methods) and active learning to strategically explore the underrepresented regions of chemical space, thereby improving model generality and guiding experiments with confidence.

Photostability and temporal degradation: A critical aspect for the practical deployment of fluorescent materials, which current ML models often overlook, is photostability, particularly photobleaching—the irreversible loss of fluorescence intensity under prolonged illumination. Predicting this time-dependent property requires datasets that include fluorescence intensity decay kinetics under standardized conditions, which are currently scarce. Future efforts should focus on incorporating such temporal stability metrics into training data. By doing so, ML models can learn to predict not only the initial brightness, but also the operational lifespan of a fluorophore, thereby guiding the design of materials with enhanced photostability for long-term applications in bioimaging and solid-state lighting.

Mechanism interpretability and physics priors: A persistent concern is the black-box nature of many ML predictions, which often lack alignment with known photophysical mechanisms. Purely data-driven models might achieve good accuracy in predicting, say, quantum yields or emission wavelengths, but they provide little insight into why a particular AIEgen is bright or how a doped phosphor’s structure affects its thermal quenching. Across systems from TADF organics to rare-earth phosphors and QDs, interpretability is key for researcher trust and knowledge generation. Incorporating physics-based priors and mechanistic knowledge into models is a promising path: for example, using descriptors grounded in quantum chemistry, enforcing physically motivated constraints (symmetry, energy conservation, selection rules), or coupling ML with first-principles calculations to ensure outputs remain physically plausible. In parallel, adopting explainable AI techniques and inherently interpretable architectures can help extract structure–property relationships (e.g., highlighting functional groups or crystal features crucial for luminescence), bridging the gap between statistical predictions and chemical understanding.

Inverse design and closed-loop pipelines: Beyond prediction, the future lies in leveraging ML for inverse design—proposing new luminescent materials with targeted properties—and integrating these models into autonomous discovery loops. Designing optimal emitters is inherently multi-objective: an ideal material must simultaneously satisfy criteria such as high quantum efficiency, specific emission color, stability, and manufacturability. Achieving this for disparate systems (from designing new AIE molecular frameworks or TADF chromophores, to optimizing dopant combinations in inorganic phosphors or perovskites) is challenging due to the enormous search space and trade-offs between objectives. Tackling it will require advanced generative models and multi-objective optimization algorithms that can navigate complex design landscapes and suggest candidates balancing competing properties. Equally important is the development of closed-loop experimental pipelines, where ML models iteratively guide high-throughput synthesis and characterization. In such a loop, an algorithm could propose promising new compositions or structures, have them rapidly synthesized and tested (for example, via automated or high-throughput experiments), then update itself with the results. This active, closed-loop approach accelerates optimization by continuously learning from experiments, and it has the potential to dramatically shorten development cycles for next-generation luminophores.

Reproducibility and benchmarking: As the field matures, issues of reproducibility and fair benchmarking of ML models have come to the forefront. Currently, studies on luminescent materials often use proprietary datasets or custom evaluation metrics, making it difficult to compare results across different investigations or material classes. A predictive model for OLED emitter efficiency developed by one group, for instance, might not be directly comparable to another group’s model for QD brightness due to differing data scales and validation protocols. The lack of common benchmarks and open repositories not only hinders reproducibility, but can also lead to overfitting on idiosyncratic datasets without the checks of external validation. Going forward, the community should prioritize the creation of shared datasets and public leaderboards for tasks like predicting emission wavelengths, quantum yields, or stability under standardized conditions. Establishing consensus performance metrics and requiring the release of code and data alongside publications will ensure that new algorithms are evaluated on a level playing field. Such efforts would greatly enhance transparency, allow for rigorous comparison of methodologies, and ultimately drive more reliable progress in ML-guided luminescent materials research.

Outlook: Addressing the above challenges will demand a coordinated, forward-looking strategy. Future machine learning frameworks for luminescent materials must be calibrated, physics-informed, and inherently multi-objective so that they not only make accurate predictions, but also provide confidence levels, obey physical laws, and optimize the trade-offs critical in real-world applications. Crucially, these advanced models should be embedded in interoperable, standardized pipelines that connect data curation, model training, and experimental validation in a seamless workflow. By coupling intelligent algorithms with rich domain knowledge and robust data infrastructure, the field can usher in a new era of luminescent materials discovery—one where AI-driven systems rapidly pinpoint innovative emitters with superior performance and do so in a transparent, reproducible, and efficient manner.

Concluding remarks for experimentalists: For researchers seeking to integrate ML into fluorescent material discovery, key practical insights emerge from this review: (1) Prioritize high-quality, standardized data curation (unifying experimental conditions, eliminating outliers, and supplementing missing information). Data quality directly determines the upper limit of model performance and serves as the core prerequisite for reliable predictions. (2) Start with interpretable models such as random forests and XGBoost to explore physicochemical correlations between structure and performance (e.g., effects of functional groups and crystal fields). After accumulating domain insights, gradually adopt complex “black-box” architectures to avoid blind predictions without mechanistic understanding. (3) Use active learning or Bayesian optimization to select candidates with “high information value”. Replace blind trial-and-error with “strategic experiments” to maximize information gain from each synthesis and characterization cycle, reducing ineffective costs. (4) Finally, foster close collaboration between experimental and computational teams to effectively embed domain knowledge into the ML workflow, ensuring that predictions are both accurate and scientifically plausible.

## Figures and Tables

**Table 1:** Available datasets for machine learning in fluorescent materials.

Dataset Name	Description	Access
PhotochemCAD	A comprehensive collection of absorption and fluorescence spectra for diverse organic fluorophores.	https://www.photochemcad.com/ (accessed on 28 September 2025)
Deep4Chem	A large-scale dataset of molecular structures paired with optical properties (absorption/emission wavelengths, quantum yields) in various solvents.	https://www.deep4chem.com/ (accessed on 28 September 2025)
CrabNet	A powerful tool for predicting inorganic material properties. While not exclusively for phosphors, it can be repurposed with phosphor data.	https://pypi.org/project/crabnet/ (accessed on 28 September 2025)
The Materials Project	A vast database of computed properties for inorganic materials. Provides band structures, formation energies, and more for potential host lattices.	https://next-gen.materialsproject.org (accessed on 28 September 2025)
Perovskite Database	This database covers data on the device performance, stability, and other aspects of perovskite solar cells, and provides interactive tools (such as band gap analysis and stability visualization).	https://perovskitedatabase.com (accessed on 28 September 2025)
NIST Atomic Spectra Database	Authoritative data on atomic emission and absorption lines. Crucial for characterizing and calibrating instruments involving rare-earth dopants.	https://www.nist.gov/pml/atomic-spectra-database (accessed on 28 September 2025)

## Data Availability

No new data were created or analyzed in this study. Data sharing is not applicable to this article.
